# Cellular function and pathological role of ATP13A2 and related P-type transport ATPases in Parkinson's disease and other neurological disorders

**DOI:** 10.3389/fnmol.2014.00048

**Published:** 2014-05-27

**Authors:** Sarah van Veen, Danny M. Sørensen, Tine Holemans, Henrik W. Holen, Michael G. Palmgren, Peter Vangheluwe

**Affiliations:** ^1^Laboratory of Cellular Transport Systems, Department of Cellular and Molecular Medicine, KU LeuvenLeuven, Belgium; ^2^Department of Plant and Environmental Sciences, Centre for Membrane Pumps in Cells and Disease – PUMPkin, University of CopenhagenFrederiksberg, Denmark

**Keywords:** alpha-synuclein, mitochondria, mitophagy, lysosome, dystonia, parkinsonism, flippase, heavy metal toxicity

## Abstract

Mutations in *ATP13A2* lead to Kufor-Rakeb syndrome, a parkinsonism with dementia. ATP13A2 belongs to the P-type transport ATPases, a large family of primary active transporters that exert vital cellular functions. However, the cellular function and transported substrate of ATP13A2 remain unknown. To discuss the role of ATP13A2 in neurodegeneration, we first provide a short description of the architecture and transport mechanism of P-type transport ATPases. Then, we briefly highlight key P-type ATPases involved in neuronal disorders such as the copper transporters ATP7A (Menkes disease), ATP7B (Wilson disease), the Na^+^/K^+^-ATPases ATP1A2 (familial hemiplegic migraine) and ATP1A3 (rapid-onset dystonia parkinsonism). Finally, we review the recent literature of ATP13A2 and discuss ATP13A2's putative cellular function in the light of what is known concerning the functions of other, better-studied P-type ATPases. We critically review the available data concerning the role of ATP13A2 in heavy metal transport and propose a possible alternative hypothesis that ATP13A2 might be a flippase. As a flippase, ATP13A2 may transport an organic molecule, such as a lipid or a peptide, from one membrane leaflet to the other. A flippase might control local lipid dynamics during vesicle formation and membrane fusion events.

## Introduction

Neurodegenerative diseases, the fourth leading cause of death in developed countries, are characterized by progressive loss of neurons within the central nervous system leading to motor and cognitive dysfunction. Alzheimer's disease (AD) and Parkinson's disease (PD) are the most common neurodegenerative disorders (Lees et al., 2009; Tolleson and Fang, 2013). Their prevalence is increasing as a consequence of the ageing population and lack of successful treatments. PD is a progressive movement disorder characterized by severe loss of dopaminergic neurons in the *substantia nigra pars compacta* (Lees et al., 2009). As a consequence of cell death, dopamine content is reduced in the basal ganglia, leading to the motor symptoms observed in patients. The cardinal symptoms of PD are resting tremor, muscle rigidity (stiffness of limbs), bradykinesia (slowness of movements) and postural instability (gait or balance problems) (reviewed in Jankovic, 2008; Lees et al., 2009; Tolleson and Fang, 2013).

A second hallmark of PD is the accumulation of aggregated α-synuclein into Lewy bodies (LBs) (Polymeropoulos et al., 1997). Moreover, mutations in the *SNCA* (α-synuclein) gene were found to be associated with the familial cases of early-onset Parkinson's disease (Spillantini et al., 1997). α-synuclein is able to form amyloid fibrils, β-sheet structures prone to aggregation, which is its main pathogenic feature. α-synuclein overexpression results in endoplasmic reticulum (ER) stress, vesicle trafficking defects, impairment of the ubiquitin-proteasome system and mitochondrial dysfunction (reviewed in Auluck et al., 2010; Bendor et al., 2013).

α-synuclein is mainly found at the presynaptic terminals of neurons (Maroteaux et al., 1988). In presynaptic terminals, α-synuclein interacts with the membranes of synaptic vesicles and associated proteins, where it appears to be a critical regulator of vesicle dynamics at the synapse (reviewed in Auluck et al., 2010; Bendor et al., 2013). It acts as a trafficking partner of synaptobrevin II (sybII) (Gordon and Cousin, 2014). At this location, α-synuclein facilitates the entry of sybII into SNARE complexes, which is a key step in the exocytotic fusion of synaptic vesicles with the presynaptic terminal (Burre et al., 2010; Gordon and Cousin, 2014). The acidic C-terminal of α-synuclein interacts with sybII whereas its N-terminal membrane-associated region is an inducible amphipathic α-helix that obtains its structure only after contact with the membrane (Auluck et al., 2010; Bendor et al., 2013). The amphipathic helix does not enter the membrane bilayer, but aligns itself parallel to the bilayer axis. Amphipathic α-helices are found in several proteins that regulate membrane vesicle trafficking and it is becoming increasingly clear that they function as membrane curvature sensors (Drin et al., 2007; Drin and Antonny, 2010; Jensen et al., 2011). Synucleins have been shown to both induce and sense membrane curvature (Middleton and Rhoades, 2010; Varkey et al., 2010; Pranke et al., 2011), which can have a significant impact on the basal fusogenic properties of synaptic vesicles.

α-synuclein aggregates accumulate in PD and are cleared via various routes, mainly including the ubiquitin-proteasome system, autophagy and lysosomal degradation pathways (Webb et al., 2003; Cuervo et al., 2004). Besides the accumulation of misfolded proteins, PD is further associated with mitochondrial dysfunction generating reactive oxygen species (ROS) and oxidative stress (Ayala et al., 2007; Auluck et al., 2010). These phenomena mutually affect each other as diseased mitochondria generate more ROS, which in turn exacerbates protein folding defects. Thus clearance of dysfunctional or damaged mitochondria and proper functioning of the protein quality control are essential for neuronal fitness and survival, but are impaired in PD (Devine et al., 2011; Jin and Youle, 2012; Tofaris, 2012; Dehay et al., 2012a). Protein quality control depends on both the proteasome and lysosome. The lysosome mediates end-stage degradation of obsolete or damaged cytoplasmic material, including protein aggregates and organelles such as mitochondria, through autophagy pathways (Webb et al., 2003; Cuervo et al., 2004; Mak et al., 2010; Jin and Youle, 2012; Tofaris, 2012; Dehay et al., 2012a).

ATP13A2 is a late endosomal/lysosomal P5-type transport ATPase that is emerging as a critical regulator of lysosomal functions (Ramirez et al., 2006; Usenovic et al., 2012a; Dehay et al., 2012b; Tsunemi and Krainc, 2014). Mutations in the ATP13A2 gene, belonging to the PARK9 PD susceptibility locus, lead to the Kufor-Rakeb syndrome (KRS), a severe early-onset autosomal recessive form of PD with dementia (Ramirez et al., 2006). Overexpression of ATP13A2 suppresses α-synuclein toxicity. This links two genetic risk factors of PD, *i.e.* ATP13A2 and α-synuclein, highlighting the central role of ATP13A2 in PD (Gitler et al., 2009). Loss of ATP13A2 function is also associated with neuronal ceroid lipofuscinosis, a lysosomal storage disorder (Farias et al., 2011; Bras et al., 2012; Schultheis et al., 2013).

This review will focus on ATP13A2 as an orphan member of the family of P-type transport ATPases. P-type ATPases are a large family of evolutionarily related primary transporters present in Archaea, Bacteria and Eukarya (reviewed in Kuhlbrandt, 2004; Palmgren and Nissen, 2011). They use the energy derived from ATP hydrolysis to transport various substrates, ranging from ions to lipids, across biological membranes against their concentration gradients. P-type ATPases are crucial for the generation of electrochemical gradients, which fuel vital cellular processes, such as secondary transport, excitability, vesicular transport and osmotic balance.

In the following sections we will give a short description of the architecture and transport mechanism of classical P-type transport ATPases. Then, we will provide an overview of those P-type ATPases that are implicated in neuronal disorders. Finally, we will review the recent literature of ATP13A2 and use available knowledge on P-type ATPase functions to discuss ATP13A2's putative cellular function and pathological role in PD.

## The family of P-type ATPases

### General features of P-type ATPases

P-type ATPases are biological pumps omnipresent in all forms of life, which are recognized by several conserved signature motifs associated with their catalytic mechanism (Axelsen and Palmgren, 1998). The main characteristic of all P-type ATPases is the formation of an acid-stable aspartyl phosphate intermediate during the catalytic cycle (hence the name P-type). The phosphorylated Asp residue is located in a highly conserved DKTG sequence motif located in the cytoplasmic part of the proteins. The events of auto-phosphorylation and auto-dephosphorylation are tightly coupled to substrate binding, transport and release.

According to sequence comparison and phylogenetic analysis, the P-type transport ATPase family can be classified into five distinct subfamilies (P1–P5), which can be further divided into additional subgroups (A, B, etc.) (Axelsen and Palmgren, 1998) (reviewed in Kuhlbrandt, 2004; Palmgren and Nissen, 2011). Importantly, the phylogenetic division correlates well with differences in the preferred transport substrates. The P1-P3 ATPases are well-characterized ion pumps: the P1A are part of bacterial K^+^ transport systems, the P1B transport heavy metals, the P2A and P2B are Ca^2+^ pumps, the P2C Na^+^/K^+^- and H^+^/K^+^-pumps are found in animals, the P2D are Na^+^ pumps in fungi and mosses and the plasma membrane H^+^ pumps of P3A are present in fungi and plants. The P3B corresponds to a small group of bacterial Mg^2+^ transporters. In contrast to inorganic ion transport, P4 ATPases participate in lipid flipping across membranes, generating membrane curvature and exposing or removing relevant signaling lipids. The substrate specificity of the last subfamily, the P5 ATPases, has yet to be revealed.

**Figure 1:**
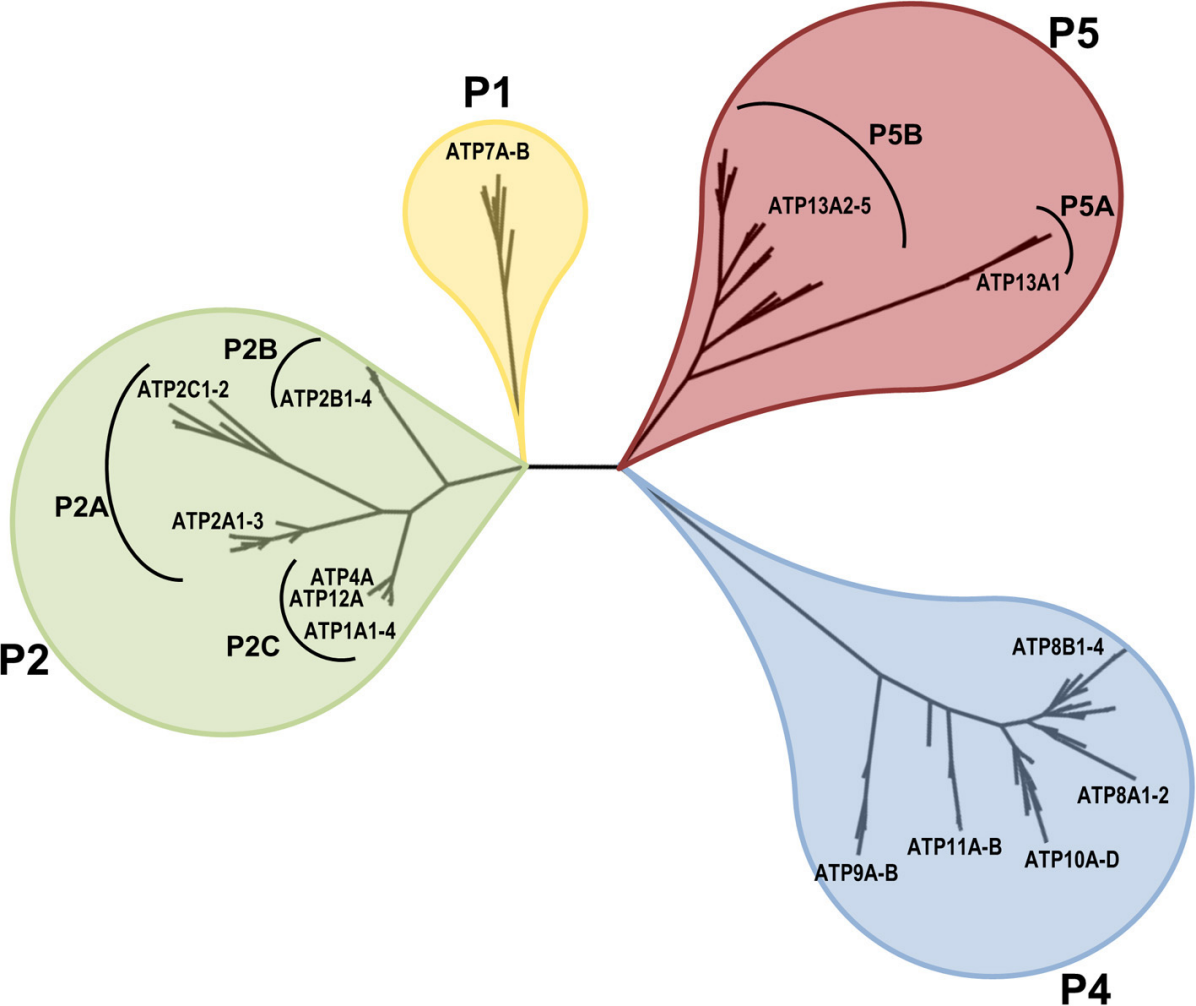
**Phyologenetic tree of the human P-type ATPases**. Phylogenetic tree based on the core protein sequences of 137 animal homologues of the 36 human P-type ATPase isoforms. ATP13A2 homologues were obtained from the database Homologene http://www.ncbi.nlm.nih.gov/homologene. Core protein sequences were generated according to the methodology described in Axelsen and Palmgren (1998). The 36 human P-type ATPases are indicated. Of note, only animal isoforms are depicted, so the P3A-type ATPases, which are uniquely found in fungi and plants and the small class of bacterial Mg^+^-ATPases of the P3B group and bacterial pumps belonging to P1A are not represented. The phylogenetic tree was rendered using www.phylogeny.fr (Dereeper et al., 2008, 2010).

Based on the conserved P-type ATPase motifs, 36 human genes are recognized and annotated in databases to encode for P-type ATPases. These include 2 copper-ATPases, 4 Na^+^/K^+^-ATPases, 2 H^+^/K^+^-ATPases, 9 Ca^2+^-ATPases, 14 putative lipid flippases and 5 P5-type ATPases with unknown substrate specificity (ATP13A1-5). Figure 1 displays the phylogenetic relationship of these 36 human P-type ATPases and their orthologs in key animal model organisms. Many P-type ATPases display broad expression profiles and fulfill many housekeeping functions, while the expression of other P-type ATPases is restricted to specific tissues e.g., brain, heart, skeletal muscle, etc.

**Figure 2:**
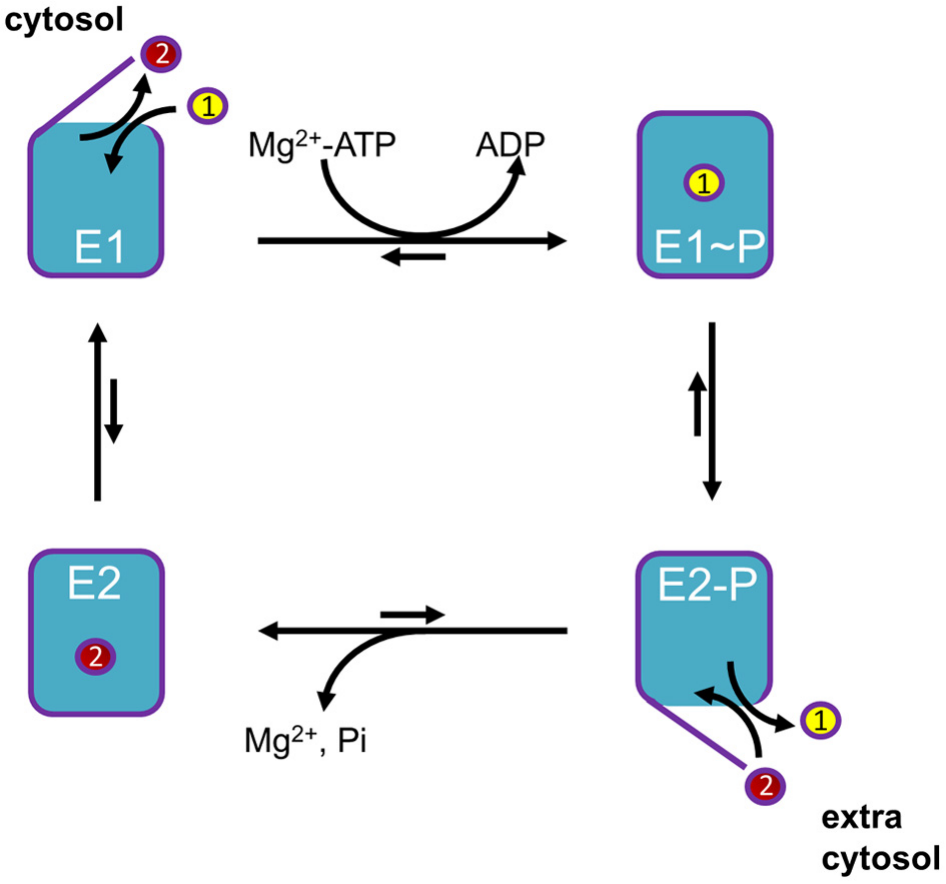
**General Post-Albers reaction scheme for P-type ATPases**. A cytosolic ligand (yellow, transported ligand 1) is transported to the extracytosolic space, whereas an extracytosolic ligand (red, counter-transported ligand 2) is imported into the cytosol. Note that the number of ligands in each direction may vary between different P-type ATPase isoforms. In short, P-type ATPases switch between two major conformations E1, with ligand binding sites facing the cytosol, and E2, with ligand binding sites facing the extracytosolic side of the membrane. The induced fit of ligand 1 binding in E1 promotes phosphorylation by Mg^+^-ATP. In this E1~P state the ligand 1 becomes occluded. The rate-limiting E1~P to E2-P transition is accompanied by major conformational changes, reorienting the ligand-binding sites toward the extracytosolic space. This decreases the affinity of the binding site for ligand 1, whereas the affinity for ligand 2 is increased. As a result, ligand 1 is released into the extracytosolic space via an open exit pathway for ligand 1 and the counter-transported ligand 2 can enter the binding cavity. The resulting conformational changes lead to dephosphorylation of E2P and the released inorganic phosphate is expelled. The ligand 2 becomes occluded, whereupon the pump is reset to the E1 state, reducing the affinity for ligand 2. The pump can now start a new cycle.

P-type ATPases use metabolic energy (ATP) to actively pump substrates against an electrochemical gradient. To prevent backflow of transported ligand(s), P-type ATPases use an alternating access mechanism (Figure 2). After substrate binding from one side, the access pathways from both sides of the membrane are transiently closed, effectively occluding the transported ion(s) in the membrane domain, before releasing them to the other side of the membrane. In addition, the affinity toward the substrate is different at both sides of the membrane. High affinity binding occurs at the side of the membrane with low substrate availability, whereas a drop in the affinity at the other side of the membrane leads to spontaneous release. These two features allow P-type ATPases to generate steep concentration and charge gradients (Kuhlbrandt, 2004; Palmgren and Nissen, 2011).

The transport mechanism of P-type ATPases can be described by the model of Post-Albers (Albers, 1967; reviewed in Kuhlbrandt, 2004; Palmgren and Nissen, 2011). During each catalytic cycle, the pumps oscillate between four major conformations (Figure 2): E1, E1P, E2P, and E2. The E1 state displays high-affinity ion-binding sites that are exposed to the cytosol. Upon ion binding, the protein reacts with an ATP nucleotide catalyzing auto-phosphorylation generating the E1P state in which ions are occluded. Then, the protein undergoes an often rate limiting transition to the E2P state where the transport binding sites are transformed into low-affinity sites facing the extra-cytosolic side of the membrane. This releases the ion(s) and allows the binding of specific counterion(s). This binding triggers E2P auto-dephosphorylation, returning to the E2 ground state in which counter-ions are occluded. All P-type ATPases are inhibited by orthovanadate, an inorganic phosphate mimic that locks the enzyme in the E2P conformation. Finally, the transition to E1 allows release of counter-ions at the cytosolic side to re-initiate the catalytical cycle.

The transport process can be overall electrogenic if translocation occurs of an unequal amount of charges at both sides of the membrane. Examples are the Na^+^/K^+^-ATPase, which transports two Na^+^ ions out of the cell in exchange for three K^+^ ions per hydrolyzed ATP, and the SERCA Ca^2+^-ATPases, which transport two Ca^2+^ ions from the cytoplasm to the lumen of the ER/SR for two to three H^+^ in the other direction. Other P-type ATPases transport only in one direction using either the forward E1 to E1P step to bind the transported substrate (P1B copper-ATPase, P3 H^+^-ATPase) while others use the E2P step for substrate binding (P4 lipid flippases) (Kuhlbrandt, 2004; Palmgren and Nissen, 2011).

### The P-type ATPase architecture

The two archetypical members of the P-type ATPase family are the sarco(endo)plasmic reticulum (SR/ER) Ca^2+^-ATPase SERCA1a and the (α1 subunit of) Na^+^/K^+^-ATPase for which there is a wealth of structural and kinetic information. SERCA1a was the first P-type ATPase to have its structure solved at high resolution (Toyoshima et al., 2000), providing detailed insights into the overall domain organization of a P-type ATPase. Since then, several other conformational states of SERCA1a have been resolved using several inhibitors, transition analogs and nucleotides locking the protein in intermediate steps of the transport mechanism. Together with a strong biochemical characterization and extensive mutagenesis, this has culminated in a detailed description of the transport mechanism of the Ca^2+^-ATPase at atomic resolution, which provides the scaffold to understand the transport process in other P-type ATPases. For a careful discussion of this topic, the reader is referred to excellent in depth reviews and references in (Toyoshima, 2009; Moller et al., 2010; Palmgren and Nissen, 2011).

**Figure 3:**
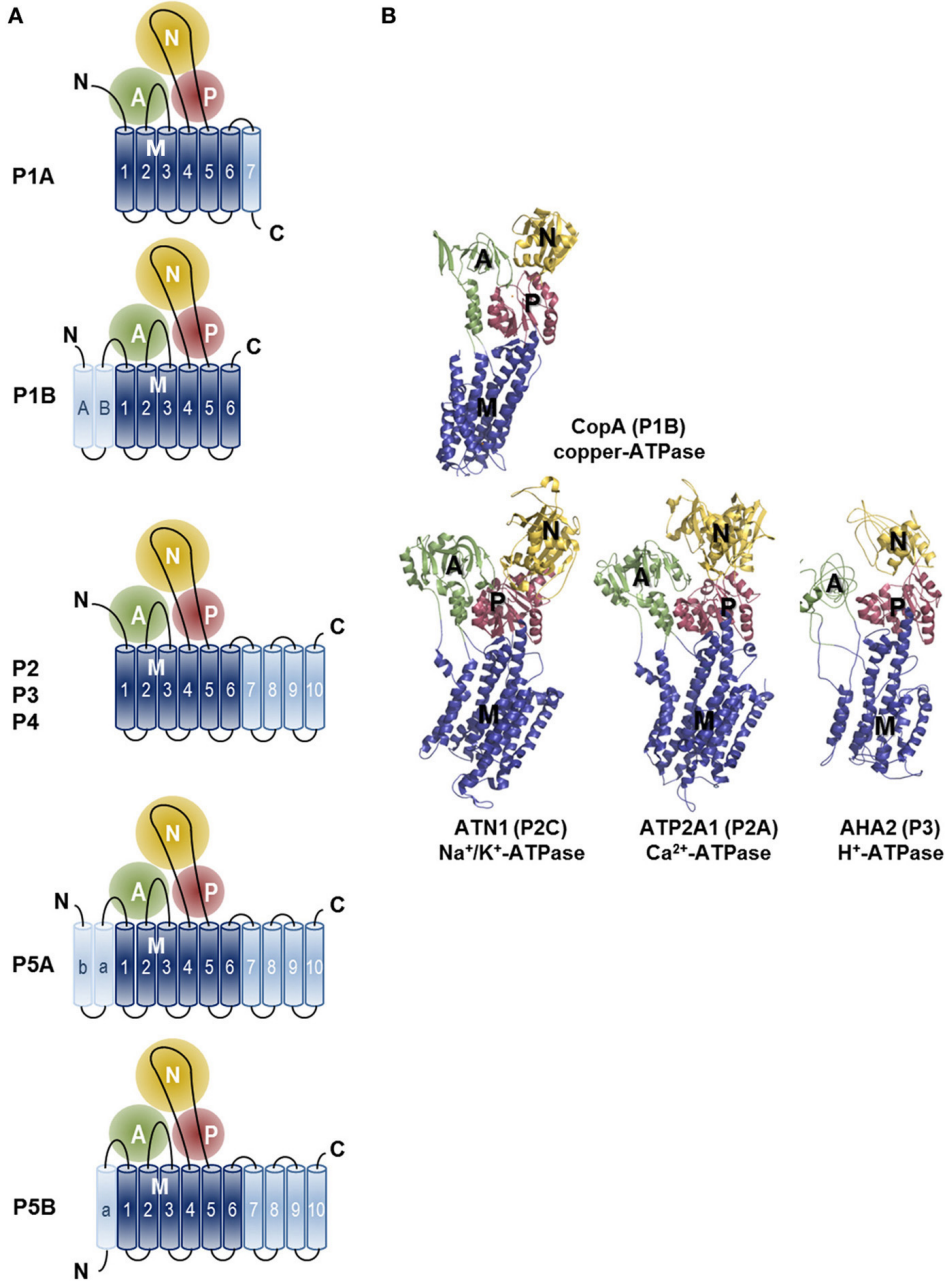
**Topology and architecture of the catalytic subunits of P-type ATPases**. **(A)** Planar topology models of the five classes of P-type ATPases (P1-P5). Nucleotide-binding domains (N, yellow), actuator domains (A, green) and phosphorylation domains (P, red) are indicated. The 6 TM helices (Polymeropoulos et al., 1997; Spillantini et al., 1997; Jankovic, 2008; Lees et al., 2009; Auluck et al., 2010; Tolleson and Fang, 2013) form the core segment of the membrane (M) domain of all P-type ATPases, which is depicted in dark blue, whereas additional N- and C-terminal helices are shown in light blue. Of note, there is one exception for the P2 ATPases, a splice variant of ATP2A2, SERCA2b, harbors an 11th TM helix at the C-terminus (not shown, Vandecaetsbeek et al., 2009). **(B)** Resolved P-type ATPase crystal structures, known up until now. The *Legionella pneumophila* CopA copper-ATPase (PDB 3RFU), a P1B-type ATPase; the rabbit P2A-type ATPase ATP2A1 (SERCA1a, PDB 2AGV), the *Squalus acanthias* Na^+^/K^+^-ATPase α-subunit ATN1 (PDB 3A3Y), a member of the P2C group and the *Arabidopsis thaliana* proton pump AHA2 (PBD 3B8C) of the P3-type ATPases. N-, A-, P- and M-domains are indicated with similar colors as in the planar models. Note that the obligatory subunits of the P1A, P2C and P4 are not shown.

Crystal structures have also been presented for other P-type ATPases including the H^+^-ATPase of plants (Pedersen et al., 2007), the Na^+^/K^+^-ATPase (Morth et al., 2007; Shinoda et al., 2009; Kanai et al., 2013; Nyblom et al., 2013) and the copper-ATPase of *Legionella pneumophila* (Gourdon et al., 2011) (Figure 3). Comparison of these structures has revealed that P-type ATPases share a strikingly similar fold despite strong sequence divergence. Four principal domains are recognized, which are conserved throughout the family: three cytoplasmic domains (nucleotide-binding, N; phosphorylation, P; actuator, A) and a transmembrane (TM) domain (M domain) (Figure 3). During the catalytic transport process the N-domain binds ATP and serves as a built-in protein kinase, which auto-phosphorylates the P-domain. The A-domain acts as an intrinsic protein phosphatase dephosphorylating the P-domain later in the catalytic cycle. The process of phosphorylation and dephosphorylation is tightly coupled to formation and deformation of high-affinity transport-binding sites in the M domain by an allosteric mechanism (Toyoshima, 2009; Moller et al., 2010; Palmgren and Nissen, 2011).

The N-domain is the least conserved cytoplasmic domain among P-type ATPases and forms the ATP binding pocket. It is situated as a large insert into the P-domain sequence stretch and is connected by a highly flexible hinge region linking the N- and P-domains (Toyoshima et al., 2000). The strongly conserved P-domain contains the P-type ATPase fingerprint with the critical Asp residue (DKTG). During each catalytic cycle, the Asp residue is alternately phosphorylated and dephosphorylated by the N-domain and the A-domain, respectively. A Lys in the nucleotide interaction site of the N-domain (KGAPE) interacts with the adenine ring of ATP delivering the γ-phosphate to the active-site residue of the P-domain. This reaction renders a high-energy aspartyl phosphate intermediate. Subsequently, the A-domain subjects the bond to hydrolysis, catalyzed by the Glu residue in the highly conserved signature motif TGE (Toyoshima, 2009; Moller et al., 2010; Palmgren and Nissen, 2011).

The M-domain, the largest of the four principal domains, comprises six to twelve α-helices (Bublitz et al., 2011) and plays a crucial role in substrate binding and transport. The cytoplasmic domains are connected to the M-domain by five flexible linker regions, four in P1-type ATPases (Palmgren and Nissen, 2011). The substrate translocation pathways are centered on the M1–M6 segments. M4 is critically important for substrate specificity and coordinating the substrate in the binding pocket. The sequence of the M4 region thus diverges between the five P-type subfamilies, corresponding to the difference in substrate specificities. M4 involves a highly conserved Pro residue, which induces unwinding of M4. This twist exposes backbone carbonyl oxygens that are used to coordinate the transported ligand (Palmgren and Nissen, 2011).

P-type ATPases also hold extended N- or C-terminal tails that regulate pump activity by intra-molecular interaction (Vandecaetsbeek et al., 2009) or via interaction of regulatory proteins (Vincenzi et al., 1980). The extensions may in addition control subcellular localization (Petris et al., 1998) or substrate delivery (Gourdon et al., 2011). Often, the N and C termini are auto-inhibitory, preventing the activation of the transporter and requiring additional stimuli for pump activation (Ekberg et al., 2010; Zhou et al., 2013).

### The P-type ATPase ion transport cycle

To translocate substrates across the membrane, P-type pumps undergo extensive conformational changes, which are driven by ATP hydrolysis. In the next section, the general catalytic mechanism of P-type ATPases will be explained based on the Ca^2+^ transport cycle of the SERCA1a pump (based on references in Toyoshima, 2009; Moller et al., 2010; Palmgren and Nissen, 2011).

#### Ca^2+^ entry and binding: E2 → E1 · 2Ca^2+^

At the start of the catalytic cycle, cytosolic Ca^2+^ ions interact with the high-affinity binding sites in the M-domain in the E1 conformation. The binding of the two Ca^2+^ ions is sequential and cooperative. The first Ca^2+^ proceeds to site I where its binding repositions a critical Asp residue on M6 (D800), which now forms the second Ca^2+^ binding site II. Upon binding of the second Ca^2+^, the gating residue E309 on M4 will capture the second Ca^2+^ ion in site II.

#### Phosphorylation and occlusion: E1 · 2Ca^2+^ → E1 ~ P · 2Ca^2+^

Via the induced fit mechanism of Ca^2+^ binding, the rearrangement of the TM helices is transmitted to the P-domain creating a Mg^2+^-binding site near the critical Asp residue (Asp^351^ in SERCA1a). Presence of Mg^2+^ is essential as this cofactor decreases the electrostatic repulsion of the γ-phosphate of ATP by the negatively charged Asp and hence, allows phosphate transfer. In this way, ATPase activity of P-type transporters in the cytosolic domains is tightly coupled to the ion binding in the M-domain, preventing unnecessary ATP hydrolysis. The transition toward the intermediate E1~ P phosphorylated state bends the P-domain and tilts the A-domain that rests on the P-domain. This exerts strain on the linkers between the A-domain and M1, M2, and M3 of the M-domain. As a result, M1-M2 is partially lifted out of the membrane forcing E309 in a fixed position, which closes the Ca^2+^ entry path (occlusion).

#### Ca^2+^ release: E1 ~ P → E2-P

Following complete γ-phosphate transfer, the ATP-mediated connection between the N- and P-domains is lost. As a consequence, the pump relaxes and the N-domain moves away from the catalytic site and stretches the linker region between the M3 helix and the A-domain. The generated tension triggers rotation of the A-domain and results in transition to the low-energy E2P state. The significant conformational changes associated with the E1P to E2P transition is the rate-limiting step in the catalytical cycle. The rearrangement of the pump opens the luminal exit pathway for Ca^2+^ by spreading out M1/M2 and M3/M4 away from M5/M6. In addition, this reduces the affinity of the Ca^2+^-binding sites promoting the luminal release of Ca^2+^. In exchange, two to three H^+^ ions bind with high affinity to the E2P state leading to occlusion of the luminal gate and further rotation of the A-domain. The A-domain rotation also brings the TGE loop closer to the phosphorylation site, shielding the aspartyl-phosphate by restricting the access of ADP or water.

#### Dephosphorylation and occlusion: E2-P → E2

The Ca^2+^-ATPase pump is reset to E1 by a series of reversal reactions leading to E2P dephosphorylation and proton countertransport. Entry of a water molecule induces a new rotation of the A-domain, which now precisely positions the Glu of the TGE-loop and the water molecule to catalyze an attack on the aspartyl phosphate. The rotation of the A-domain also repositions M1/M2 and the cation-binding site with the protons becomes occluded. Thereafter, the A-domain disengages from the phosphorylation site resulting in transition of the E2 state to the relaxed E1 conformation associated with release of the counterions.

### Domain organization and signature motifs in P5B-type ATPases

In comparison with the well-studied SERCA1a Ca^2+^ pump, little is known about the P5-type ATPases to which ATP13A2 belongs. P5-type ATPases are found in all eukaryotic genomes, but are absent in bacterial genomes (Moller et al., 2008; Sorensen et al., 2010). Based on the conservation of residues in the putative transport binding sites (Moller et al., 2008) and on their predicted TM topology (Sorensen et al., 2010), the P5 subfamily can be divided into two groups, P5A and P5B (Figures 1, 3). Exactly one P5A (ATP13A1 in humans) and at least one P5B isoform is found in all eukaryotic genomes, except for land plants, which have lost the P5B genes (Moller et al., 2008). Multiple P5B isoforms exist in higher vertebrates (four in humans, ATP13A2-5) and some invertebrate lineages (three in *Caenorhabditis elegans*, CATP-5 to 7).

The P5-ATPase membrane topology is unusual (Sorensen et al., 2010). In addition to the 10 classical TM helices, two extra TM spanning helices are predicted in the N terminus of the P5A ATPases (Ma and Mb), whereas the P5B group is marked by a single predicted N-terminal TM helix (Ma). Therefore, the P5A would consist of 12 and the P5B of 11 TM helices. The poor sequence conservation of the extra N-terminal helices suggest that they are not critical for substrate coordination during transport (Sorensen et al., 2010). Instead, as in other P-type ATPases, the N-terminal region may directly function as a regulator of catalytic function or serve as the docking site for other regulatory proteins (Gourdon et al., 2011). Of all P-type ATPases, only the P5 and the P1B heavy metal pumps contain additional N-terminal membrane-spanning segments (Figure 3). The structure of a P1B pump, the CopA copper transporter, was recently solved (Gourdon et al., 2011). The structure clearly depicts that the Mb forms an N-terminal docking platform for the binding of a copper chaperone, which delivers copper to the pump directly, or via the N-terminal heavy metal binding domains (Gourdon et al., 2011). Interestingly, also the P5 ATPases contain conserved Pro or Gly residues at a similar position in the additional N-terminal membrane helices (Mb for P5A ATPases Sorensen et al., 2010 and Ma for P5B ATPases). In analogy herewith, the extended N terminus of the P5 may follow a similar fold as in the P1B, representing a docking platform for substrate delivery.

Like other P-type ATPases, P5 isoforms, including ATP13A2, contain the key signature motifs KGAPE for ATP coordination (N-domain), DKTG for auto-phosphorylation (P-domain) and TGE for dephosphorylation (A-domain) (Kwasnicka-Crawford et al., 2005; Ramirez et al., 2006). This indicates that P5-ATPases like other P-type ATPases catalyze the hydrolysis of ATP to auto-phosphorylate the enzyme on a conserved Asp residue in the P-domain.

The presence of a highly conserved M4 region within P5 ATPases indicates that in P5-type ATPases the hydrolysis of ATP may be coupled to the transport of a ligand close to the M4 region (Moller et al., 2008; Sorensen et al., 2010). Spontaneous auto-phosphorylation has already been observed for the yeast P5A ATPase Spf1p and the plant P5A ATPase HvP5A1 (Corradi et al., 2012; Sorensen et al., 2012), but so far it remains unclear whether P5B-ATPases such as ATP13A2 also form a phospho-intermediate.

**Figure 4:**
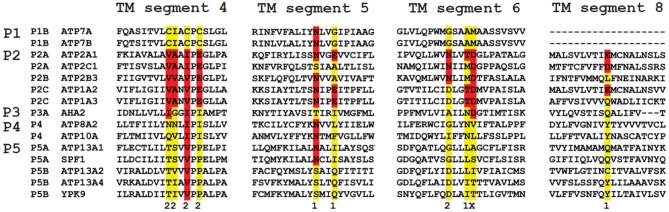
**Sequence comparison of the TM helices in P-type ATPases of various subfamilies**. The residues involved in Ca^2+^ binding in the two Ca^2+^ binding sites (site 1 and 2) in the SERCA1a Ca^2+^ pump (ATP2A1) are distributed over four TM helices: M4, 5, 6 and 8. The colored residues are part of the Ca^2+^ binding sites in ATP2A1 and numbers 1 and 2 refer to the number of the Ca^2+^-binding site to which the residue contributes (x is contributing to both site 1 and site 2). The sequence of the M4, M5, M6 and M8 helices is compared with those of the P-type ATPases that are involved in neurological disorders. Also the yeast P5 ATPases Spf1p and Ypk9p, the Ca^2+^/Mn^2+^-ATPase SPCA as well as the proton pump AHA2 are included for comparison. M4 shows the highest degree of conservation. Highlighted in red are conserved residues as compared to the ATP2A1 Ca^2+^ binding site sequence, whereas in yellow the non-conserved residues are indicated. For each subfamily, a signature motif can be recognized in M4, which corresponds well with the substrate specificity. The PPELP and PPALP sequences of P5A- and P5B-type ATPases have little in common with other P-type ATPase signature motifs, which might indicate that the transported ligand is significantly different.

The M4 segment of the P5-type ATPase corresponds to the putative substrate binding site and contains a double Pro in (PPxxP) (Moller et al., 2008; Sorensen et al., 2010). This may exacerbate the twist of the M4 segment imposed by the initial Pro, which might have a significant impact on the mechanism of substrate coordination and transport. P5A sequences contain a PP(E/D)xPx(E/D) motif, whereas P5B sequences are characterized by a PP(A/V)xP(A/V)x motif (Moller et al., 2008; Sorensen et al., 2010) (Figure 4). The negative charges in the unwound M4 helix of P5A compared to the corresponding hydrophobic residues in P5B may suggest that both subgroups display different substrate specificities (Moller et al., 2008; Sorensen et al., 2010). For this reason, we will focus only on the P5B ATPases in this review.

## P-type ATPases and neurodegeneration

P-type ATPases play important roles in the nervous system, ranging from regulation of Ca^2+^ homeostasis, osmotic balance, electrical excitability, uptake of trace elements to vesicular transport processes. It is therefore not surprising to see that loss-of-function mutations in many P-type ATPase isoforms are detrimental for neuronal functions. In the following sections we will provide a short overview of those P-type ATPases that according to genetic information are implicated in neurological disorders (Table 1).

**Table 1:** **P-type ATPases involved in neuronal disorders**.

**P-type**	**Gene**	**Substrate**	**Disorder**	**Ref**.
P1B	ATP7A	Cu^+^	Menkes disease (MD)	[OMIM:309400]
			Occipital horn syndrome (OHS)	[OMIM:304150]
			Spinal muscular atrophy, distal, X-linked 3 (SMAX3)	[OMIM:300489]
P1B	ATP7B	Cu^+^	Wilson disease (WD)	[OMIM:277900]
			Possible genetic risk factor for Alzheimer's disease (AD) and parkinsonism	Bull et al., 1993; Telianidis et al., 2013
P2B	ATP2B3	Ca^2+^	Early onset X-linked spinocerebellar ataxia 1	[OMIM:300014]
P2C	ATP1A2	Na^+^/K^+^	Familial hemiplegic migraine type 2 (FHM2)	[OMIM:602481]
			Alternating hemiplegia of childhood 1 (AHC1)	[OMIM:104290]
P2C	ATP1A3	Na^+^/K^+^	Rapid-onset dystonia parkinsonism (DYT12, RDP)	[OMIM:128235]
			Alternating hemiplegia of childhood 2 (AHC2)	[OMIM:614820]
P4	ATP8A2	PS	Cerebellar ataxia, mental retardation and disequilibrium syndrome 4 (CAMRQ4)	[OMIM:615268]
P4	ATP10A	?	Angelman syndrome (AS)	Blanco-Arias et al., 2009
P5B	ATP13A2	?	Kufor-Rakeb syndrome (KRS)	[OMIM:606693]
			Neuronal ceroid lipofuscinosis (NCL)	
P5B	ATP13A4	?	Specific language impairment (SLI)	Gourdon et al., 2011; Lohmann and Klein, 2013
			autism spectrum disorders (ASD)	Ugolino et al., 2011

OMIM entries are shown for the most well established genetic disorders. References are shown for disorders that have been linked genetically to mutations in the indicated gene(s).

### P1B-type ATPases in neurological disorders

The P1B-type ATPase subfamily consists of the two copper-transporting isoforms in human, ATP7A and ATP7B. ATP7A is ubiquitously expressed in all tissues, including the brain, but except the liver, regulating homeostatic maintenance of cell copper levels. ATP7B is highly expressed in the liver. Mutations in ATP7A are associated with Menkes disease (MD) (Chelly et al., 1993; Mercer et al., 1993; Vulpe et al., 1993), while ATP7B mutations cause Wilson's disease (WD) (Bull et al., 1993) (reviewed in Gupta and Lutsenko, 2009; Kaler, 2011; Telianidis et al., 2013).

#### ATP7A

Mutations in *ATP7A* are associated with MD, an X-linked recessive disorder characterized by progressive neurodegeneration and connective tissue dysfunction (Chelly et al., 1993; Mercer et al., 1993; Vulpe et al., 1993). The clinical manifestations of MD include severe seizures associated with cerebral atrophy, vascular abnormalities, kinky hair structure, hypopigmentation, growth retardation and death in early childhood. These features originate from a generalized copper deficiency that triggers dysfunction of several cuproenzymes (Kaler, 2011). *ATP7A* mutations also underlie occipital horn syndrome (OHS), a milder disease with moderate neurologic symptoms and prominent connective tissue disturbances (Kaler, 2011). Recently, a novel ATP7A-related disease phenotype was discovered, spinal muscular atrophy, distal, X-linked 3 (SMAX3), which is characterized by atrophy of the lower limb muscles (Kennerson et al., 2010).

ATP7A is targeted to the trans-Golgi network from where it supplies copper to the copper-dependent enzymes as they migrate through the secretory pathway. Under conditions of elevated copper, ATP7A relocalizes to the plasma membrane where it promotes the efflux of copper from cells (Petris et al., 1998). To date, over 500 disease-causing variations of *ATP7A* have been reported, mostly substitutions and deletions (ATP7A database, www.LOVD.nl/ATP7A) which lead to misfolding (Kim et al., 2002), impaired copper-induced trafficking (Kim et al., 2003) or reduced copper-ATPase activity (Paulsen et al., 2006).

#### ATP7B

WD is an autosomal recessive disorder caused by mutations in *ATP7B* (Bull et al., 1993), a copper-ATPase which is mainly expressed in liver and implicated in biliary copper excretion (Telianidis et al., 2013). ATP7B dysfunction results in the toxic buildup of copper in liver. Brain copper accumulation develops secondary to liver disease and leads to degeneration of the basal ganglia. As a consequence, WD patients present movement disorders such as tremor, dystonia and parkinsonism (Machado et al., 2006).

A number of single nucleotide polymorphisms in ATP7B are associated with an increased risk of AD (Squitti et al., 2013). Furthermore, it has been suggested that a single mutated ATP7B allele may confer susceptibility for (late-onset) parkinsonism (Sechi et al., 2007).

### P2-type ATPases in neurological disorders

The P2-type ATPases constitute the best characterized subfamily of P-type ATPases. The human P2 isoforms can be subdivided into three groups, P2A, P2B and P2C (P2D is not represented in humans, but consists of eukaryotic Na^+^-ATPases). The P2A group contains the well-known SERCA and Secretory Pathway/Golgi (SPCA) Ca^2+^-ATPases, whereas the plasma membrane Ca^2+^-ATPases (PMCAs) belong to the P2B-ATPases. The P2C-subgroup encompasses the Na^+^/K^+^-ATPases and the gastric H^+^/K^+^-pumps (Kuhlbrandt, 2004; Palmgren and Nissen, 2011).

#### Na^+^/K^+^-ATPase

The Na^+^/K^+^-ATPase generates vital Na^+^ and K^+^ gradients over the plasma membrane by expelling three Na^+^ ions in exchange for two K^+^ ions. This is essential for many physiological functions in the nervous system such as cell volume control, the drive of secondary active transport systems and the support of electrical excitability (reviewed in Benarroch, 2011). The α-subunit is the catalytical subunit of the Na^+^/K^+^-ATPase that exists in four isoforms (ATP1A1-4 or α1-4), which display tissue specific and developmental dependent expression. Only the *ATP1A1-3* genes are expressed in the nervous system. The α-subunit forms a hetero-oligomer with a β- and γ-subunit. β is critical for proper targeting and affects the K^+^ affinity (Hasler et al., 2001), whereas γ (belonging to the FXYD family) mainly regulates the Na^+^ affinity of the pump (Geering, 2005).

The neurological disorders familial hemiplegic migraine type 2 (FHM2), alternating hemiplegia of childhood (AHC), and rapid-onset dystonia parkinsonism (RDP) are autosomal dominant disorders caused by mutations of the Na^+^/K^+^-ATPase α2 (FHM2 and AHC1) and α3 (AHC2 and RDP) isoforms. α2 (ATP1A2) is primarily expressed in astrocytes and drives Na^+^-dependent Glu uptake and removes excess K^+^ from the extracellular space during neuronal excitation. α3 (ATP1A3) is predominantly expressed in neurons and is involved in post-stimulus recovery (reviewed in Brashear et al., 2014).

***ATP1A2***. *ATP1A2* mutations lead to FHM2, a severe subtype of migraine with aura and temporary hemiparesis. More than 20 mutations in the α2-subunit are known to cause FHM2 (De Fusco et al., 2003; Jurkat-Rott et al., 2004; Schack et al., 2012). A disturbed clearance of extracellular K^+^ by glial cells underlies FHM2, which is related to an impaired pumping rate (Schack et al., 2012). *ATP1A2* mutations may also underlie basilar migraine (BM), which is a subtype of migraine with aura originating from the brainstem or involvement of both hemispheres (Ambrosini et al., 2005). In addition, a mutation in the *ATP1A2* gene was also identified in affected members of a family with AHC1 (Swoboda et al., 2004). FHM2, BM, and AHC1 are allelic disorders with overlapping phenotypes.

***ATP1A3***. Mutations in *ATP1A3* (also known as dystonia-12, DYT12) lead to RDP, which is a rare autosomal dominant movement disorder with variable penetrance, characterized by the abrupt start of dystonia with signs of parkinsonism (de Carvalho Aguiar et al., 2004). The onset of RDP (at the age of 4–55) is often triggered by physical or emotional stress, fever, childbirth, or alcohol consumption. RDP-associated mutations are predominantly located in highly conserved residues in the TM domain of ATP1A3, which mainly affect the Na^+^ affinity (Rodacker et al., 2006; Blanco-Arias et al., 2009). The resulting intracellular Na^+^ increase may possibly affect the Na^+^/Ca^2+^ exchange system and subsequently lead to increased intracellular Ca^2+^ impacting on Ca^2+^-dependent signaling pathways, such as neurotransmitter release (Rodacker et al., 2006). Mutations in *ATP1A3* are also implicated in AHC2 (Heinzen et al., 2012), which generally has earlier onset than RDP and is characterized by transient episodes of hemiplegia often shifting from one side of the body to the other. AHC2 and RDP present overlapping clinical features such as dystonia with a bulbar preference (Heinzen et al., 2012; Brashear et al., 2014).

According to the crystal structure of Na^+^/K^+^-ATPase, the C-terminal tail is inserted within a binding pocket between TM helices (Morth et al., 2007). This tail controls Na^+^ and proton binding at the third Na^+^ site. At least eight disease mutations occur in this C-terminal ion pathway (Poulsen et al., 2010). Two disease mutations have been reported in which the C terminus is extended by one Tyr residue in a patient with RDP (Blanco-Arias et al., 2009) and by a 28-residue long segment in a patient with FHM2 (Jurkat-Rott et al., 2004).

#### ATP2B3

PMCA isoforms (ATP2B1-4) remove Ca^2+^ from the cytosol to the extracellular environment. *ATP2B3* mutations cause early onset X-linked spinocerebellar ataxia-1, a disorder characterized by degeneration of the cerebellum (Zanni et al., 2012). Clinical manifestations include hypotonia at birth, dysarthria, gait ataxia difficulty standing, slow eye movements and delayed motor development. PMCA3 is highly expressed in the cerebellum, a critical region for motor coordination (Zanni et al., 2012).

### P4-type ATPases in neurological disorders

The human genome encodes 14 P4-type ATPases, which are putative lipid flippases involved in aminophospholipid transport across membrane bilayers. P4-type ATPases are the first class of P-type transporters that do not transport inorganic ions (reviewed in Graham, 2004; Poulsen et al., 2008; van der Mark et al., 2013).

#### ATP8A2

The P4-type ATPase ATP8A2 is a phosphatidylserine (PS) translocase, which is localized to the plasma membrane and highly expressed in retina and brain, particularly in the cerebellum. The P4 lipid flippase ATP8A2 is involved in localization of PS to the inner leaflet of the plasma membrane (Zhu et al., 2012). A missense mutation located in a M domain of ATP8A2 is associated with cerebellar ataxia, mental retardation and dysequilibrium syndrome (CAMRQ), an autosomal recessive disorder characterized by dysarthric speech and cerebellar atrophy with or without quadrupedal gait (Onat et al., 2013). Mice carrying loss-of-function mutations in the *Atp8a2* gene develop axonal degeneration resulting in progressive ataxia and neurodegeneration (Zhu et al., 2012). Loss of ATP8A2 disrupts PS asymmetry which might lead to fragile neuronal membranes that are more prone to degeneration. Defective vesicular trafficking may provide an alternative explanation (Zhu et al., 2012).

#### ATP10A

ATP10A is a putative aminophospholipid translocase. *ATP10A* maps within the most common interval of deletion (15q11-q13) leading to Angelman syndrome (AS). This syndrome is marked by neurobehavioral anomalies that include severe mental retardation, ataxia and epilepsy. AS patients with imprinting mutations or with maternal deletions of 15q11-q13 display little or no ATP10A expression (Meguro et al., 2001).

### P5-type ATPases in neurological disorders

Two members of the P5-type ATPases are implicated in neurological disorders. Little is known about the substrate specificity and cellular function of P5-type ATPases, but their putative function is extensively discussed in section 5 of this review.

#### ATP13A2

Loss-of-function mutations in ATP13A2 (PARK9) are a known cause of Kufor-Rakeb syndrome (KRS), an autosomal recessive disorder characterized by juvenile-onset Parkinsonism associated with dementia (Ramirez et al., 2006). This syndrome was first described in 1994 in a consanguineous family originating from Kufor-Rakeb, Jordan (Najim al-Din et al., 1994). Today, several homozygous and compound heterozygous mutations have been described that result in truncation of the ATP13A2 protein leading to loss-of-function (Ramirez et al., 2006; Schneider et al., 2010; Crosiers et al., 2011; Park et al., 2011). The clinical phenotype of KRS comprises pyramidal degeneration, supranuclear gaze palsy and severe cognitive decline (Williams et al., 2005). Brain MRI of KRS patients revealed generalized atrophy and putaminal and caudate iron accumulation, classifying KRS amongst neurodegeneration with brain iron accumulation (Bruggemann et al., 2010; Schneider et al., 2010), although others reported KRS patients without iron accumulation (Chien et al., 2011). KRS can be classified as a complex dystonia, i.e., a form of dystonia that occurs in conjunction with other neurological or non-neurological symptoms (Lohmann and Klein, 2013; Klein, 2014).

Whereas wild-type ATP13A2 is localized to late endosomal and lysosomal membranes (Ramirez et al., 2006), the truncating KRS mutations lead to retention of the protein in the ER resulting in ER stress and proteasomal degradation via the ER-associated degradation pathway (Ugolino et al., 2011). Other missense mutations in ATP13A2 have been identified that are associated with early-onset Parkinsonism (Di Fonzo et al., 2007; Lin et al., 2008; Ning et al., 2008; Santoro et al., 2011). Similar to KRS mutations, homozygous missense mutations disrupt normal localization and function of ATP13A2 while heterozygous missense mutations may impair ATPase activity (Podhajska et al., 2012). Moreover, ATP13A2 protein levels are increased in surviving neurons of humans with PD/dementia with LBs indicative of a putative protective function of high levels of ATP13A2. In LBs positive neurons ATP13A2 did not co-localize directly, but rather surrounds the LBs (Ramonet et al., 2012).

In dogs (Farias et al., 2011; Wohlke et al., 2011) and mice (Schultheis et al., 2013), loss of ATP13A2 elicits neuronal ceroid lipofuscinosis (NCL), a lysosomal storage disorder characterized by the accumulation of autofluorescent lipopigment. The phenotype of NCL partially overlaps with that of KRS and involves dysarthria, cerebellar ataxia, rigidity, bradykinesia and cognitive impairment. *Atp13a2* deficient mice exhibit age-dependent sensorimotor deficits that resemble motor symptoms observed in KRS, NCL and PD patients. Moreover, loss of Atp13a2 leads to lipofuscin accumulation and α-synuclein aggregation in the hippocampus, critical features of NCL and PD, respectively (Schultheis et al., 2013). Also a homozygous ATP13A2 missense mutation was reported that is associated with juvenile NCL in humans (Bras et al., 2012). Extensive lipofuscinosis was demonstrated in neuronal and glial cells of cortex, basal ganglia and cerebellum (Bras et al., 2012). These findings underline the importance of ATP13A2 in the lysosomal pathway for α-synuclein degradation and suggest that lysosomal dysfunction might represent a link between lipofuscinosis and α-synuclein accumulation.

#### ATP13A4

ATP13A4 has been linked to language delay (Kwasnicka-Crawford et al., 2005; Worthey et al., 2013) and autism spectrum disorders (ASD) (Vallipuram et al., 2010). In two patients, disruption of ATP13A4 led to specific language impairment characterized by delayed expressive and receptive language, without further cognitive deficiencies (Kwasnicka-Crawford et al., 2005). Moreover, in a Finnish genome-wide screen for ASD, an autism susceptibility locus was identified on chromosome 3q25-27, nearby *ATP13A4* (Auranen et al., 2002). In six study participants, a Glu646Asp sequence variant was found, which is located between the fourth and fifth TM region of ATP13A4, near the conserved Asp residue and the N-domain (Kwasnicka-Crawford et al., 2005).

There is very little knowledge concerning the biological role of ATP13A4. In mice, Atp13a4 is mainly expressed in stomach and brain (Schultheis et al., 2004). Atp13a4 expression varies throughout all regions of the adult mouse brain, with the highest relative expression in cerebellum. Atp13a4 expression is also developmentally regulated, peaking at late neurogenesis, suggesting a function in neuronal development (Vallipuram et al., 2010; Weingarten et al., 2012). In humans, ATP13A4 mRNA has been detected in multiple organs, with relatively low expression in the brain, where expression was observed in the lateral inferior frontal cortex (Broca's area) and the temporoparietal cortex (Wernicke's area) (Kwasnicka-Crawford et al., 2005), areas of importance for language output and input, respectively. ATP13A4 is observed in the ER membrane and increases intracellular Ca^2+^ levels when overexpressed in COS-7 cells (Vallipuram et al., 2010). The intracellular Ca^2+^ increase is not observed in cells overexpressing the Glu646Asp variant, implying that the substitution might impair the ability of ATP13A4 to regulate Ca^2+^ transport (Vallipuram et al., 2010).

## Cellular function of P5B ATPases in model organisms

A major bottleneck in unraveling the role of ATP13A2 in neurological disorders is the fact that virtually nothing is known concerning the molecular function and substrate specificity of the P5B-type ATPases. In this section, we will discuss the cellular role of ATP13A2 orthologs in different model organisms.

### YPK9 in the yeast *saccharomyces cerevisiae*

**Figure 5:**
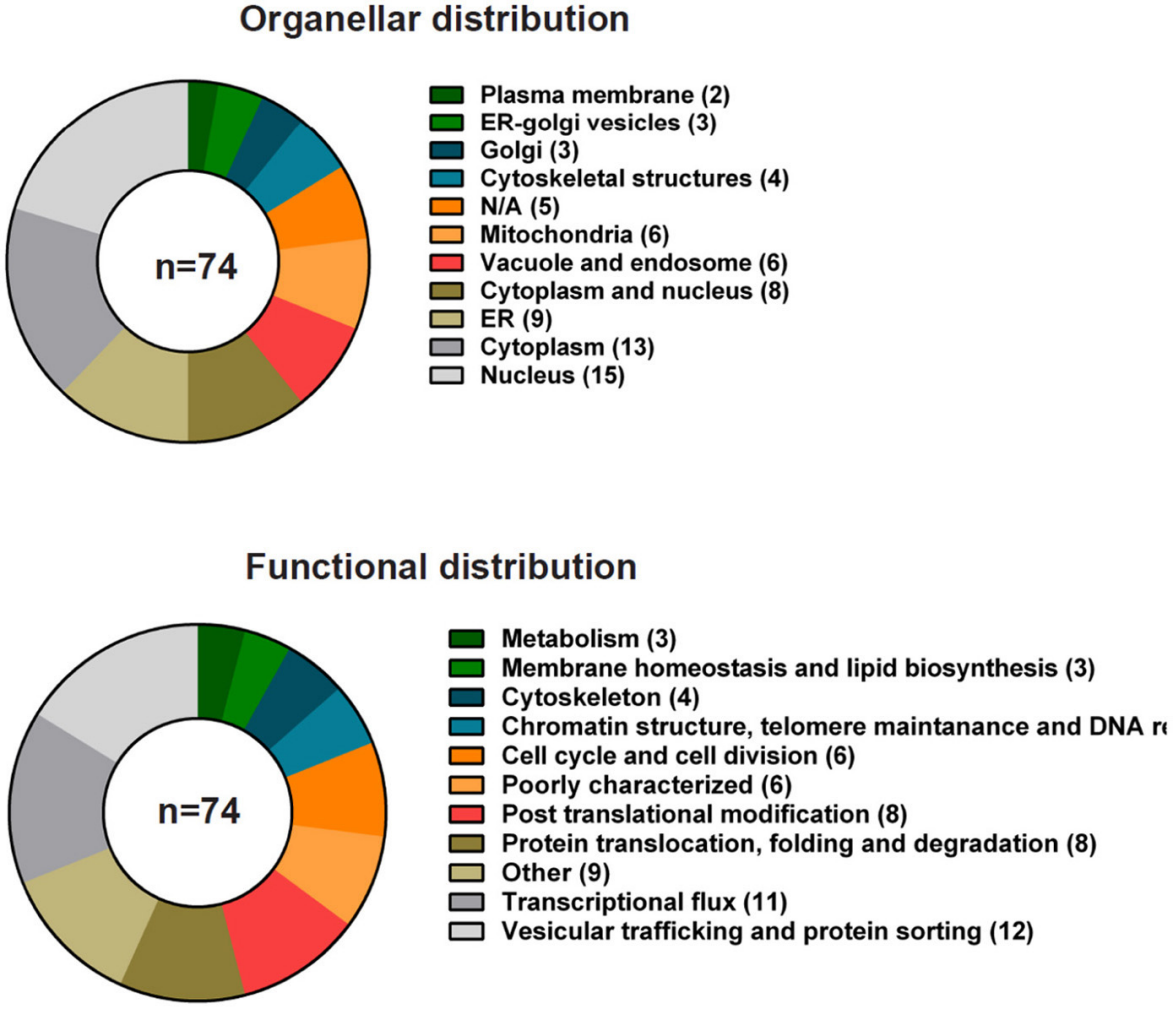
**Genetic interactions of *YPK9* in *S. cerevisiae* grouped according to cellular localization and function**. Summary of the known genetic interactions of the yeast ATP13A2 ortholog *YPK9*. The data were collected from the yeast genome database (http://yeastgenome.org, see Supplemental Table 1) and were classified using BioGrid (http://thebiogrid.org) according to organellar distribution and function.

Ypk9p (yeast PARK9), is the single P5B-type ATPase in the yeast *Saccharomyces cerevisiae* and resides in the vacuolar membrane, the yeast equivalent of the mammalian lysosome (Gitler et al., 2009). Studies in yeast show that Ypk9p is involved in protecting cells against Mn^2+^ toxicity (Gitler et al., 2009) and, more broadly, heavy metals (Schmidt et al., 2009; Chesi et al., 2012; Kong et al., 2014), but the ion specificity might be strain dependent (Schmidt et al., 2009). Little or no other phenotypes have been reported. According to the *S. cerevisiae* genome database (http://yeastgenome.org) 74 genes or gene products have been reported as interactors of *YPK9*. Most interactions are genetic (72 in total) highlighting genes that when deleted in the absence of *YPK9* result in synthetic growth effects or phenotypic suppression or enhancement (Figure 5). Supplemental Table 1 integrates data on interactions from high- and low-throughput studies in yeast available at BioGrid (http://thebiogrid.org). The interactors vary both with respect to cellular location and predicted function. Surprisingly, only a few of the interactors are located in the endosome and vacuolar systems (6 out of 74 interactors). The most prevalent interactions participate in vesicular trafficking and protein sorting (12 out of 74 interactors) and transcriptional flux (11 out of 74) (Figure 5).

Only one gene, TATA-binding protein-associated factor-1, *TAF1*, has so far been reported to be synthetic lethal in combination with deletion of *YPK9*. *TAF1* encodes the largest transcription factor TFIID subunit involved in RNA pol II transcription initiation, promoter binding and G1/S progression (Walker et al., 1997). Taf1p relocalizes to the cytosol in response to hypoxia, whereas subsequent oxygen exposure restores the nuclear localization (Dastidar et al., 2012). Also the expression of ATP13A2 in mammalian cells is upregulated in conditions of hypoxia (Xu et al., 2012). In humans, *TAF1* is associated with complex dystonia (X-linked parkinsonism, unconfirmed genetic evidence) (Makino et al., 2007) (reviewed in Lohmann and Klein, 2013; Klein, 2014). This might suggest a putative link between ATP13A2 and TAF1 in humans. Human TAF1 possesses protein kinase, ubiquitin-activating and -conjugating activities and histone acetyltransferase activities. These activities control transcription of genes involved in the G1 progression in mammalian cells such as cyclin D1 and cyclin A (Kloet et al., 2012).

*OPI3* and *YME1* are so far the only two positive genetic interactors that have been identified. Thus, deletion of any of these genes in combination with a deletion of *YPK9* alleviates growth effects caused by loss of either. *OPI3* encodes the methylene-fatty-acyl-phospholipid synthase, which catalyzes the last two steps in phosphatidylcholine biosynthesis at the ER membrane in contact zones with the plasma membrane (Tavassoli et al., 2013). *YME1* encodes the catalytic subunit of the i-AAA protease complex that is responsible for degradation of unfolded mitochondrial proteins in the intermembrane space. Yme1p mediates Atg32p processing, which is required for mitophagy (Wang et al., 2013). It also contributes substantially to the proteolytic turnover of phosphatidylserine decarboxylase-1 (PSD1) located at the mitochondrial inner membrane (Nebauer et al., 2007).

The link between *YPK9* and the mitochondria is further underscored by negative genetic interactions with six other genes (*ATP5, MDL2, MMM1, FMT1, GEP3*, and *OXR1*) that encode proteins related to mitochondrial function. Negative interactions are defined as genes that upon deletion, in combination with a *YPK9* deletion, display aggravated growth effects as compared to the loss of either individual gene. The six genes are related to maintenance of mitochondrial homeostasis (*ATP5, MDL2, MMM1*), mitochondrial biogenesis (*FMT1, GEP3*) and resistance to oxidative damage (*OXR1*). Several interactors of *YPK9* can thus be related to mitochondrial function, which substantiates a link between *YPK9* and protection from oxidative stress.

The remaining majority of interactors related to *YPK9* have been identified in a genome-wide screen aimed at defining mechanisms by which *YPK9* protects cells from Mn^2+^ toxicity in yeast (Chesi et al., 2012). At physiological conditions, *YPK9* genetically interacts with essential genes involved in the cell cycle (*APC5, CDC28, CDC53, SFH1, POL3, CDC11* and *CDC12*), cellular transport and vesicular trafficking (*ALG1, GAB1, BET2* and *MYO2*) or RNA processing (*DIM1*). Cdc11p and Cdc12p belong to the septin family, which includes highly conserved GTP-binding proteins found in eukaryotes. Septins provide a scaffold to support cell division, polarity and compartmentalization and have been implicated in diverse neurodegenerative disorders in humans, including in PD and α-synuclein mediated toxicity (Hall and Russell, 2004). *BET2* encodes the beta subunit of type II geranylgeranyl transferase (Rossi et al., 1991) that is required for vesicular transport between the ER and the Golgi (Newman et al., 1990). Bet2p provides a membrane anchor to the Rab-like protein Ypt1p, which like Ypk9p, protects yeast cells from α-synuclein toxicity (Cooper et al., 2006).

Yeast genes involved in Mn^2+^ protection mainly belong to categories of vesicle-mediated transport, vacuolar organization and chromatin remodeling (Chesi et al., 2012). *YPK9* deletion modifies Mn^2+^ tolerance of a subset of these genes. Interactors that increase Mn^2+^ sensitivity of *YPK9* are implicated in vacuolar and vesicle organization and membrane fusion (such as *VAM3, VAM6, SWF1*, and *GLO3*), whereas interactors that increase Mn^2+^ tolerance (such as *SIF2, HMO1, LEO1, APQ12*, and *MOG1*) seem to be involved in chromatin organization, histone modification and nuclear transport (Chesi et al., 2012). However, it is unclear why none of the established Mn^2+^ or heavy metal transport systems have been reported to be genetically linked to *YPK9*. This could be because *YPK9* is located upstream of established transport systems that take care of Mn^2+^ clearance. Ypk9p might for instance be implicated in the regulation of vesicular transport routes that control Mn^2+^ homeostasis. As Mn^2+^ ions are redox active Ypk9p may also regulate the removal pathway of damaged mitochondria via autophagy.

Taken together, the genetic interaction data from yeast suggest a potential role for *YPK9* in the cell cycle and vesicular trafficking in combination with proper mitochondrial function.

### Animal models of ATP13A2

#### C. elegans

*Caenorhabditis elegans* contains three P5B genes, *catp-5* to *7*. In contrast to the vacuolar/lysosomal localization of other P5B ATPases, CATP-5 locates to the plasma membrane at the apical side of intestinal cells (Heinick et al., 2010). *catp-5* mutant strains are impaired in polyamine uptake (Heinick et al., 2010). Polyamines are ubiquitous cellular components that affect numerous biological processes such as cell cycle progression. Polyamines interact with anionic binding sites of macromolecules such as nucleic acids and phospholipids. *catp-5* might either encode a polyamine transporter or the gene might positively regulate polyamine uptake (Heinick et al., 2010). In mammalian CHO cells, ATP13A2 overexpression leads to a two-fold higher accumulation of the polyamine spermidine. It was shown that the ATP-dependent spermidine uptake was increased in a lysosomal and late endosomal fraction further supporting the notion that the ATP13A2 protein mediates polyamine uptake (De La Hera et al., 2013). The higher polyamine uptake rate might further explain the increased cytotoxic effects of paraquat, a toxic polyamine analogue that is an environmental risk factor for PD (Pinto Fde et al., 2012).

CATP-6 locates depending on the tissue type to either cytoplasmic punctae likely corresponding to vesicles associated with the lysosome or to the plasma membrane (Lambie et al., 2013). *catp-6*, was identified in a RNAi screen for genes stabilizing synthetic α-synuclein, representing a putative functional homologue of ATP13A2 (Hamamichi et al., 2008). The *catp-6* locus genetically interacts with *gon-2* and *gem-1* (Lambie et al., 2013). *gon-2* encodes a TRPM cation channel protein that is required for Mg^2+^ uptake, whereas *gem-1* encodes for the SLC16A transporter, which might be a putative monocarboxylate transporter. It was suggested that the *catp-6* gene product governs Mg^2+^ uptake by regulating the trafficking of transporters or other regulatory proteins to the plasma membrane (Lambie et al., 2013).

#### Mouse

The phenotype of a genetic knock-out mouse model of *Atp13a2* was recently described (Schultheis et al., 2013). The insertion site of the *Neo* gene in the genome of the *Atp13a2*^−/−^ mice would still allow the formation of a truncated and mutated N-terminal Atp13a2 fragment consisting of the first 341 amino acids of Atp13a2 followed by 168 unrelated amino acids. Although the mutated transcript is clearly formed, no traces of the mutated protein were detected, suggesting that it may either be unstable or inefficiently translated (Schultheis et al., 2013).

As mentioned above, the *Atp13a2*^−/−^ mice show α-synuclein accumulation as occurs in PD and related synucleinopathies, and accumulation of lipofuscin deposits, characteristic of NCL (Schultheis et al., 2013). The α-synuclein aggregation occurred predominantly in the hippocampus, but not in the cortex or cerebellum. Also the expression of some genes involved in PD is altered between the *striatum* and *substantia nigra* in *Atp13a2*^−/−^ vs. *Atp13a2*^+/+^ mice (Schultheis et al., 2013).

Twenty to twenty nine months old *Atp13a2*^−/−^ mice perform more poorly on several sensorimotor tests. More specifically, the aged *Atp13a2*^−/−^ mice display impaired aspects of motor learning. The gait analysis revealed a shortened stride length. Also reduced hindlimb stepping, impairments in fine motor skills and orofacial movements involved in nest building were reported (Schultheis et al., 2013). These deficits are similar to those observed in other genetic mouse models of PD and ataxia and also resemble aspects of motor dysfunction observed in KRS, NCL, and PD (Schultheis et al., 2013). Cognitive function and emotional reactivity were also changed in *Atp13a2*^−/−^ mice. Mutant mice demonstrated greater exploratory behavior without changes in general locomotor activity or anxiety. Because the behavioral phenotype was not detectable until old age, some compensatory mechanisms might take place, which might be related to other members of the P5-ATPase family. But so far, no evidence was found for a compensatory upregulation of other P5-type ATPases (Schultheis et al., 2013).

#### Zebra fish

In contrast, knocking out *ATP13A2* in the zebra fish results in severe retardation already at the embryonic stage of development (Lopes da Fonseca et al., 2013). As the mouse genome contains four P5B genes (*Atp13a2-5*), while the zebra fish only one (*ATP13A2*), the late-onset mouse phenotype associated with loss of *ATP13A2* would suggest that some of the P5B homologues in mice may be functionally redundant, possibly compensating for each other's loss (Schultheis et al., 2013). Further studies will be required to shed light on the possible redundancy of P5B function and whether compensatory effects from other P5 alleles can take place.

## Cellular roles of human ATP13A2

### ATP13A2 expression profile

*ATP13A2* is mapped to the PARK9 PD susceptibility locus on chromosome 1p36. ATP13A2 is predominantly expressed in the brain, particularly in the dopaminergic neurons of the *substantia nigra* (Ramirez et al., 2006). Studies in mouse have shown that Atp13a1 (P5A) and Atp13a2 (P5B) are broadly expressed in many tissues with the highest expression of Atp13a2 observed in the brain. Atp13a4 and Atp13a5 (both P5B) are only expressed in brain and stomach while Atp13a3 (P5B) has a wider expression pattern that includes brain and other internal organs like colon, kidney and liver (Schultheis et al., 2004). It thus seems that all P5-type ATPase isoforms are expressed in the brain although individual members express at different levels during various developmental stages. Expression of Atp13a2 peaks during neurogenesis while Atp13a5 peaks at the adult stage (Weingarten et al., 2012). These observations are in line with a significant, yet undescribed role for P5B ATPases in brain development and function.

At least three ATP13A2 splice variants are reported (Ugolino et al., 2011). Variant 1 is the longest and counts 1180 amino acids. Variant 2 contains a five amino acid in-frame deletion in the N-terminus (1175 amino acids), whereas variant 3 is 1158 amino acids long and appears to be an anomalous protein. Here, the last two TM helices are replaced by an unusual sequence stretch and variant 3 also lacks an important part of the connection of the TM region with the cytosolic domains. Based on comparison with other P-type ATPases these alterations will probably have a significant impact on enzymatic activity. Variant 3 is retained in the ER and is rapidly degraded, questioning whether it serves a cellular role (Ugolino et al., 2011).

The promoter region of the human ATP13A2 gene contains hypoxia response elements, which can bind to the transcription factor hypoxia inducible factor 1a (HIF-1a). Hypoxic conditions up-regulate transcription of the *ATP13A2* gene in both HEK293 and dopaminergic MN9D cells (Xu et al., 2012). Also Mn^2+^ and Zn^2+^ elevate ATP13A2 expression in several cell lines (see further details below) (Tan et al., 2011; Tsunemi and Krainc, 2014).

### Intracellular localization of ATP13A2

The general accepted view is that ATP13A2 is targeted to acidic compartments, *i.e.* the late endosomes and lysosomes, because of a co-localization with LAMP1/2a, Rab7, and Lysotracker. Also the loss of ATP13A2 leads to lysosomal dysfunction and an increase in the size and number of the lysosomes (Dehay et al., 2012b; Usenovic et al., 2012a). Originally, it was suggested that ATP13A2 is localized to late endosomes/lysosomes based on overexpression studies of tagged ATP13A2 (Ramirez et al., 2006). However, this view was recently challenged (Kong et al., 2014). In both differentiated SHSY5Y cells and rat primary neurons, the endogenous ATP13A2 associates closely together with LC3, a marker of the autophagosomes. More specifically, the authors concluded that the endogenous ATP13A2 occupies the outer limiting membrane of multivesicular bodies (MVBs), a morphologically distinctive late endosome compartment. MVBs undergo dynamic rearrangements and sorting of lipids and proteins via inward budding of the membrane generating multiple intra-luminal vesicles (ILVs). By fusing with the plasma membrane, MVBs can release the ILVs in the extracellular space as exosomes. MVBs can also fuse with autophagosomes from autophagy pathways producing hybrid structures referred to as amphisomes, which then can fuse with lysosomes for cargo degradation. ATP13A2 is also observed in the outer membrane of amphisomes (Kong et al., 2014).

### ATP13A2 is involved in autophagy and mitochondrial clearance

Mitochondrial dysfunction is tightly linked to the pathogenesis of PD (Auluck et al., 2010; Jin and Youle, 2012; Gautier et al., 2014). Strong support comes from the observations that 1-methyl-4-phenyl-1,2,3,4-tetrahydropyridine (MPTP), a potent mitochondrial complex I inhibitor, triggers a PD-like syndrome (Burns et al., 1983; Langston and Ballard, 1983). Several PD-associated genes, namely parkin, PINK1 and DJ-1 play a role in mitochondrial dynamics and clearance strengthening the concept that mitochondrial dysfunction and the production of ROS are consistent features of PD (Auluck et al., 2010; Jin and Youle, 2012). Thus, clearance of dysfunctional or damaged mitochondria and misfolded proteins is essential for neuronal fitness and survival (Jin and Youle, 2012; Gautier et al., 2014) and the lysosome is a vital organelle for this quality control (Jin and Youle, 2012; Tofaris, 2012; Dehay et al., 2013). Organelles, cytoplasmic material and protein aggregates are delivered to the lysosome via various autophagy pathways. Macroautophagy (or just autophagy) involves the formation of double-layered membrane autophagosomes, which encapsulate cytoplasmic materials for delivery to the lysosomes for degradation. Mitochondria are removed through a specific autophagy pathway called mitophagy (Jin and Youle, 2012). Soluble proteins can also be selectively degraded in the lysosome through uptake by the lysosomal receptor LAMP2a, via a process known as chaperone-mediated autophagy (CMA) (Mak et al., 2010). Micro-autophagy involves the direct engulfment of cytoplasmic material by invagination of the late endosomal/lysosomal membrane. Whereas the function of micro-autophagy in mammalian cells is unknown, both macro-autophagy and CMA are key processes in neurodegeneration and α-synuclein removal (Webb et al., 2003; Mak et al., 2010) (reviewed in Xilouri and Stefanis, 2011).

Studies in KRS patient-derived fibroblasts and ATP13A2-deficient cell lines have revealed that mutations of ATP13A2 or knockdown of the gene transcript lead to several lysosomal alterations. First the number and size of lysosomes is increased (Usenovic et al., 2012a). Also lysosomal dysfunction is reported involving impaired lysosomal acidification, decreased proteolytic processing of lysosomal enzymes, reduced degradation of lysosomal substrates (e.g., α-synuclein) and impaired lysosomal-mediated clearance of autophagosomes (Dehay et al., 2012b; Usenovic et al., 2012a; Tsunemi and Krainc, 2014).

In parallel, a strong link between ATP13A2 and mitochondrial dysfunction is emerging. Loss of ATP13A2 function impairs mitochondrial maintenance and leads to oxidative stress. ATP13A2 expression protects mammalian cells toward mitochondrial and oxidative stress (Covy et al., 2012). In fibroblasts of patients with non-functional ATP13A2 ATP production rates are decreased. Also a higher frequency of mitochondrial DNA lesions, increased oxygen consumption rates and increased fragmentation of the mitochondrial network have been observed (Grunewald et al., 2012).

A role for ATP13A2 in autophagy and mitochondrial clearance has been suggested, but mechanistic details are lacking (Grunewald et al., 2012; Gusdon et al., 2012; Park et al., 2014). ATP13A2 regulates mitochondrial bioenergetics through macroautophagy. ATP13A2 knockdown reduces the autophagic flux in SHSY5Y cells (Gusdon et al., 2012) and lysosomal-mediated clearance of autophagosomes was impaired in patient-derived ATP13A2^−/−^ fibroblasts (Dehay et al., 2012b) and in ATP13A2 knockdown neurons (Usenovic et al., 2012a). Several physical interactors of ATP13A2 are involved in mitophagy whereas others are involved in protein quality, protein sorting, vesicular transport and membrane fusion (Usenovic et al., 2012b). Evidence from genetic interaction studies in yeast also provides a link between *YPK9* and mitochondrial clearance (see above).

The fact that ATP13A2 expression is upregulated under oxidative stress (Xu et al., 2012) seems to indicate that ATP13A2 is particularly important in conditions of oxidative stress, for instance arising from mitochondrial dysfunction. Defective mitochondrial clearance may also account for the increased sensitivity toward Zn^2+^ toxicity in ATP13A2^−/−^ cells (Park et al., 2014). Indeed, Zn^2+^ induces mitochondrial ROS production, which results in mitochondrial dysfunction and fragmentation (Park et al., 2014). Importantly, treatment with an antioxidant completely abolishes Zn^2+^-induced cell death in ATP13A2^−/−^ cells (Park et al., 2014). The link between Zn^2+^ and mitochondrial stress is further emphasized by the observation that Zn^2+^ potentiates, whereas Zn^2+^ chelation protects against MPTP-induced PD (Sheline et al., 2013). Along the same lines, loss of ATP13A2 and impaired mitochondrial clearance may explain the observed intolerance toward Mn^2+^ (Gitler et al., 2009; Schmidt et al., 2009) and paraquat (Pinto Fde et al., 2012). Since polyamines can function as ROS scavengers, the increased uptake of polyamines (De La Hera et al., 2013) may protect ATP13A2^−/−^ cells toward oxidative stress, although high levels of polyamines are also toxic.

Together, these observations suggest that ATP13A2 controls mitochondrial maintenance, which would lend further support to converging lysosomal and mitochondrial pathways in PD pathogenesis (Jin and Youle, 2012; Tofaris, 2012; Dehay et al., 2013). By controlling the autophagy-lysosomal activity ATP13A2 may serve an essential function by removing damaged or dysfunctional proteins and organelles.

### ATP13A2 is involved in vesicular transport

The long list of putative physical interactors (Usenovic et al., 2012b) points into the direction of ATP13A2 as a scaffolding protein in the regulation of vesicular processes. Like other P-type ATPases, such as the Na^+^/K^+^-ATPase (Xie and Xie, 2005) and the SERCA2 Ca^2+^-ATPase (Vangheluwe et al., 2005), ATP13A2 might be acting as a scaffold and exert a transporting function at the same time. Regulation of vesicular processes may include *de novo* vesicle formation, vesicular transport, vesicular sorting mechanisms and vesicle fusion. *E.g.* ATP13A2 directly interacts with several components of the SNARE complex that is involved in vesicle docking and fusion (Usenovic et al., 2012b). A function in vesicular transport can be easily reconciled with the established roles of ATP13A2 in mitochondrial clearance (Gusdon et al., 2012; Park et al., 2014) and α-synuclein removal (Gitler et al., 2009; Kong et al., 2014), which depend on autophagy pathways. In addition, ATP13A2 controls vesicle-dependent Zn^2+^ and α-synuclein removal mechanisms through exosomes, which are ILVs formed in the MVBs that fuse with the plasma membrane (Kong et al., 2014). Genetic evidence in yeast also provides a link between *YPK9* and vesicular transport (see above).

### The connection between ATP13A2 and α-synuclein

ATP13A2 protects cells toward α-synuclein toxicity. This has been observed in several model systems including yeast, *C. elegans* and mammalian cells (Gitler et al., 2009). Like α-synuclein, ATP13A2 might be implicated in vesicle trafficking and mitochondrial dysfunction. Several hypotheses may explain the protective effect of ATP13A2.

- By regulating lysosomal functions ATP13A2 might control lysosomal α-synuclein degradation and prevent the build-up of α-synuclein aggregates (Dehay et al., 2012b; Usenovic et al., 2012a; Tsunemi and Krainc, 2014).
- Alternatively, ATP13A2 may control the delivery of α-synuclein to the lysosomes by regulating different autophagy pathways such as macro-autophagy (Dehay et al., 2012b; Usenovic et al., 2012a) and chaperone-mediated autophagy, two autophagic routes that control α-synuclein turnover (Webb et al., 2003; Mak et al., 2010).
- α-synuclein may prevent the membrane fusion of the mitochondria resulting in increased mitochondrial fragmentation (Kamp et al., 2010). α-synuclein also impairs mitochondrial function (Auluck et al., 2010). The protective effect of ATP13A2 toward α-synuclein toxicity might therefore be related to the positive effect of ATP13A2 on mitochondrial appearance (Grunewald et al., 2012; Gusdon et al., 2012; Park et al., 2014), which might compensate excessive mitochondrial fragmentation. One can speculate that via promotion of mitochondrial clearance, ATP13A2 might provide protection toward α-synuclein-induced mitochondrial fragmentation.
- ATP13A2 promotes the removal of α-synuclein out of the cell via exosomes reducing the α-synuclein stress in cells (Kong et al., 2014).
- As α-synuclein interacts with membranes and the amount of α-synuclein interaction with the membrane seems to correlate with the degree of toxicity (Auluck et al., 2010; Kuwahara et al., 2012), it is a tempting hypothesis that ATP13A2 might affect α-synuclein membrane interactions.

### ATP13A2 controls cellular ion homeostasis

ATP13A2 causes protection toward several heavy metals (Gitler et al., 2009; Schmidt et al., 2009; Kong et al., 2014) and in KRS patients iron deposits in the brain are observed (Bruggemann et al., 2010; Schneider et al., 2010). The prevailing hypothesis is therefore that ATP13A2 is a lysosomal cation pump (Gitler et al., 2009). This hypothesis is furthermore based on sequence similarity with other P-type ATPases (Ramirez et al., 2006), among which most transport cations, e.g., to generate vital electrochemical ion gradients, to relocalize essential elements or dispose toxic metal ions.

## What is the transported ligand—if any—of ATP13A2?

Although P5B ATPases have essentially all sequence requirements to act as transporters, their ligands so far remain unidentified. ATP13A2 may regulate lysosomal function by transporting ions (Gitler et al., 2009) or an essential co-factor required for lysosomal enzyme activity (Covy et al., 2012). ATP13A2 might also take up metal ions in lysosomes or MVBs/exosomes to remove excess ions (Kong et al., 2014). The possibility that ATP13A2 works closely together with the V-type ATPases to pump protons to contribute to the low pH in the late endosome/lysosome can also not be excluded (Dehay et al., 2012b). In this section we will critically review the prevalent concept that ATP13A2 is a cation transporter by comparing sequence characteristics of ATP13A2 with better described P-type ion pumps and discussing the available physiological and biochemical evidence for transport of proposed ligands. Finally, we discuss the possibility that ATP13A2 might pump organic ions, such as lipids or peptides from one membrane leaflet to the other.

### Cation(s) possibly transported by ATP13A2

ATP13A2 has been linked to Mn^2+^, Zn^2+^, Mg^2+^, and H^+^ homeostasis suggesting that ATP13A2 might be implicated in the transport of these cations.

#### Is ATP13A2 a Mn^2+^ transporter?

ATP13A2 was first suggested to be a lysosomal Mn^2+^ transporter (Gitler et al., 2009). Mn^2+^ is a biologically relevant metal that functions as a cofactor of many enzymes, such as carboxylases and phosphatases in the cytosol, sugar transferases and sulfatases in the Golgi and the mitochondrial superoxide dismutase SOD2 (Vangheluwe et al., 2009; Tuschl et al., 2013). Little is known about Mn^2+^ requirements in lysosomes or MVBs, but Mn^2+^ uptake in the MVBs and lysosome could constitute a Mn^2+^ detoxification pathway to remove excessive Mn^2+^. This would prevent Mn^2+^ toxicity, which evokes extrapyramidal syndromes resembling PD and dystonia (Vangheluwe et al., 2009; Tuschl et al., 2013). Mn^2+^-induced cell death involves oxidative stress, interference with Ca^2+^ and iron homeostasis, DNA damage and mitochondrial dysfunction (Tan et al., 2011). It is thus clear that Mn^2+^ levels need to be properly controlled, but the responsible pathways remain incompletely understood.

A role of ATP13A2 in Mn^2+^ homeostasis and Mn^2+^ toxicity has been proposed based on the following observations. Loss of *YPK9* in yeast leads to an increased sensitivity toward Mn^2+^ (Gitler et al., 2009; Schmidt et al., 2009), whereas mammalian cells (HEK293, Neuro2a and NLF neuroblastoma cells) that overexpress ATP13A2 showed resistance to MnCl_2_-induced cytotoxicity (Tan et al., 2011; Covy et al., 2012). Atomic absorption spectrophotometry further revealed that HEK293 cells overexpressing ATP13A2 accumulate less Mn^2+^ when cells were pre-exposed to MnCl_2_. Also the endogenous ATP13A2 expression levels increase when HEK293 cells are exposed to MnCl_2_ (Tan et al., 2011). Thus, ATP13A2 regulates and is controlled by the intracellular Mn^2+^-concentration providing a strong link between ATP13A2 and Mn^2+^ homeostasis. Although these observations indicate that ATP13A2 is implicated in the removal pathway for Mn^2+^, the available evidence that ATP13A2 would be a Mn^2+^ transporter remains circumstantial since no direct Mn^2+^ transport or lysosomal Mn^2+^ uptake has been demonstrated.

For instance, instead of possibly transporting Mn^2+^ directly, ATP13A2 might influence other Mn^2+^ removal pathways which can depend on vesicular transport and/or other Mn^2+^ carriers. It is clear that efficient Mn^2+^ resistance in yeast depends on all steps in the secretory pathway involving proteins of vesicle-mediated transport, vacuolar organization and chromatin remodeling (Chesi et al., 2012). This suggests that Mn^2+^ removal may occur mainly via vesicular transport routes. In the brain, Mn^2+^ detoxification also depends on several members of the solute carrier (SLC) family including the proton coupled transporters SLC11A2/DMT1/NRAMP2 and SLC40A1/ferroportin, the putative Zn^2+^/Mn^2+^ transporter SLC30A10/ZnT10 and SLC39A14/ZIP14 (DeWitt et al., 2013; Tuschl et al., 2013). These proteins are involved in Mn^2+^ transport and typically carry several metal species. SLC members are involved in the transport of several divalent metal cations, such as Zn^2+^, Mn^2+^, Fe^2+^, Ni^2+^, Cu^2+^, Co^2+^, and Cd^2+^. This is also the case for the transferrin receptor (TfR) carrying Fe^2+^ and trivalent Mn^3+^ (DeWitt et al., 2013) and for the Secretory Pathway Ca^2+^/Mn^2+^-transport ATPase SPCA1/PMR1 (Vangheluwe et al., 2009). The relative importance of all these Mn^2+^ transporters or removal pathways in the brain or how ATP13A2 affects these pathways remains to be determined.

Several Mn^2+^ transport routes were studied in neuronal cells. Mn^2+^ is sequestered into the Golgi/secretory pathway compartments by SPCA1/PMR1, which also belongs to the P-type ATPase family (P2-type) (Vangheluwe et al., 2009). So far, the two SPCA isoforms SPCA1 (ubiquitous) and SPCA2 (secretory cells) are the only known P-type ATPases in animals that transport Mn^2+^ in intracellular stores with a high affinity. As such, SPCA1 might be implicated in the removal of toxic Mn^2+^ from neurons through the secretory pathway (Vangheluwe et al., 2009). However, SPCA expression levels decrease with Mn^2+^-exposure and SPCA activity is inhibited by high concentrations of Mn^2+^, suggesting that other Mn^2+^-removal mechanisms may prevail in neurons (Sepulveda et al., 2012).

The ubiquitous ZIP14 is present in the plasma membrane and promotes Mn^2+^ uptake in SHSY5Y neuroblastoma cells. Conversely, SLC30A10 controls Mn^2+^ secretion in SHSY5Y cells, which is thought to be a Mn^2+^ transporter (Quadri et al., 2012). Mutations in SLC30A10 cause extreme neurotoxic accumulation of Mn^2+^ in liver and brain triggering dystonia and parkinsonism (Quadri et al., 2012). The subcellular localization of SLC30A10 matches with different compartments, such as the Golgi system, endosomes, and the plasma membrane (Quadri et al., 2012). The human SLC30A10 complements defective Mn^2+^ uptake in yeast cells lacking the Ca^2+^/Mn^2+^ ATPase PMR1 (Tuschl et al., 2012) and the endogenous SLC30A10 expression increases with Mn^2+^ exposure in HepG2 hepatocellular carcinoma cells. These observations provide a strong link between SLC30A10 and maintaining Mn^2+^ homeostasis. Finally, neurons also take up Fe^2+^ and trivalent Mn^3+^ via DMT1 and TfR (DeWitt et al., 2013).

#### Is ATP13A2 a Zn^2+^ transporter?

Besides Mn^2+^, Ypk9p protects yeast against other heavy metals, including Zn^2+^, Cd^2+^, Ni^2+^, and Se^2+^ (Gitler et al., 2009; Schmidt et al., 2009; Kong et al., 2014). In mammalian cell systems, a link between ATP13A2 and Mn^2+^, Ca^2+^, Cd^2+^, Zn^2+^, or Ni^2+^ homeostasis has been reported. A protective effect of ATP13A2 overexpression toward Ni^2+^ and Mn^2+^ in mammalian NLF cells was described (Covy et al., 2012), whereas the knockdown of ATP13A2 increases the sensitivity of SHSY5Y cells toward Zn^2+^, but strangely not Mn^2+^. This challenges the view that ATP13A2 would be a Mn^2+^ transporter. Peptide fragments of ATP13A2 bind Mn^2+^, Zn^2+^, and copper (Remelli et al., 2013). ATP13A2 also regulates basal and Cd^2+^-induced intracellular Ca^2+^ levels in neurons (Ramonet et al., 2012).

Several recent studies underscored a strong link between ATP13A2 and Zn^2+^ homeostasis (Kong et al., 2014; Park et al., 2014; Tsunemi and Krainc, 2014). Thus, Zn^2+^ is another strong candidate ligand for ATP13A2-mediated transport. Neurons are sensitive to both Zn^2+^ deficiency and excess (Sensi et al., 2009) and Zn^2+^ levels are increased in PD (Hozumi et al., 2011). Fibroblasts carrying homozygous or compound heterozygous ATP13A2 disease mutations and also patient-derived olfactory neurospheres (hONs), mouse primary embryonic cortical neurons and SHSY5Y cells with ATP13A2 knock down are highly sensitive to Zn^2+^ exposure, whereas sensitivity to Mn^2+^ is less pronounced (Kong et al., 2014; Park et al., 2014; Tsunemi and Krainc, 2014). In addition, the expression of endogenous ATP13A2 in primary neurons was elevated in the presence of Zn^2+^ (Tsunemi and Krainc, 2014). In conditions of Zn^2+^ overload, the acidic or LC3-positive vesicles of *ATP13A2*^−/−^ cells accumulate less Zn^2+^ (Park et al., 2014; Tsunemi and Krainc, 2014). Conversely, via X-ray fluorescence microscopy total intracellular Zn^2+^ levels were estimated to be 60% higher in *ATP13A2*^−/−^ hONs than in control cells (Kong et al., 2014). This would suggest that ATP13A2 contributes to Zn^2+^ efflux from the cell.

Whether ATP13A2 pumps Zn^2+^ directly or rather affects other Zn^2+^ transporters or vesicular transport remains to be clarified. In the hONs, the majority of known secondary Zn^2+^ transporters were upregulated in *ATP13A2*^−/−^ cells, pointing to a severe Zn^2+^ dyshomeostasis. These include 9 members of the SLC30 family of Zn^2+^ transporters (ZnTs) that mediate Zn^2+^ efflux and 14 members of the SLC39 family of ZRT/IRT-related proteins (Zn^2+^ importing proteins, ZIP) that facilitate influx of Zn^2+^ (Park et al., 2014). It is unclear how much of the altered expression pattern of these Zn^2+^ transporters explains the mislocalization of Zn^2+^ in *ATP13A2* deficient cells.

Nevertheless, the result of impaired ATP13A2 activity is a rise in cytosolic Zn^2+^ concentrations. Zn^2+^ dyshomeostasis has been associated with a variety of neurological disorders (Sensi et al., 2009). This affects multiple cellular functions including the mitochondria (Park et al., 2014) and lysosomes (Tsunemi and Krainc, 2014), making it difficult to assess the underlying mechanism of ATP13A2-mediated Zn^2+^ protection. Zn^2+^ induces lysosomal dysfunction, which negatively impacts on lysosomal pH, lysosomal proteolysis and accumulation of α-synuclein (Tsunemi and Krainc, 2014). Loss of ATP13A2 also leads to lysosomal dysfunction, which potentiates the Zn^2+^-related effects (Dehay et al., 2012b; Tsunemi and Krainc, 2014).

Zn^2+^ is not involved in redox reactions and therefore does not generate oxidative stress by itself. However, excessive mitochondrial Zn^2+^ uptake in conditions of high Zn^2+^ exposure inhibits several enzymes and complexes of the mitochondria leading to the production of ROS. This imposes oxidative stress, which can induce cell death (Park et al., 2014). As mentioned above, the loss of ATP13A2 is associated with mitochondrial dysfunction, which is related to impaired mitochondrial clearance, presumably due to insufficient lysosomal degradation (Grunewald et al., 2012; Gusdon et al., 2012; Ramonet et al., 2012). The improper cytosolic removal of Zn^2+^ in ATP13A2^−/−^ cells might further impose mitochondrial stress, triggering severe ROS production (Park et al., 2014). Moreover, an increased accumulation of failing mitochondria in ATP13A2^−/−^ cells in conditions of Zn^2+^ exposure may contribute to the increased sensitivity of Zn^2+^. The extensive mitochondrial dysfunction and fragmentation may lead to ATP depletion and/or ROS production resulting in cellular degeneration. Importantly, treatment with an antioxidant completely abolishes Zn^2+^-induced cell death in ATP13A2^−/−^ cells, indicating that the ROS production during Zn^2+^-induced mitochondrial failure largely accounts for the toxic effects of Zn^2+^ (Park et al., 2014).

In conclusion, it appears that the Zn^2+^ phenotype of *ATP13A2*^−/−^ cells can largely be explained without imposing that ATP13A2 is a Zn^2+^ transporter.

#### Is ATP13A2 a Mg^2^+ transporter?

The ATP13A2 homologue Kil2 in *Dicytostelium discoideum*, a phagocytic bacterial predator, is required for Mg^2+^-dependent killing of ingested *Klebsiella* (Lelong et al., 2011). Also the ATP13A2 homologue CATP-6 in *C. elegans* has been implicated in Mg^2+^ uptake (Lambie et al., 2013). In a purified system, the ATPase activity of the P5A ATPase Spf1p in *S. cerevisiae* is stimulated by Mg^2+^ ions (Cronin et al., 2002). These observations might indicate that Mg^2+^ plays a role in both P5A and P5B ATPases. However, all P-type ATPases require Mg^2+^ for proper ATP coordination and phosphorylation of the conserved Asp in the P-domain (Moller et al., 2010). So for any P-type ATPase it is difficult to discriminate between transport or non-transport related effects of Mg^2+^ on the ATP-hydrolytic activity.

#### Is ATP13A2 a H^+^ transporter?

ATP13A2 is present in acidic compartments. Because loss of ATP13A2 leads to an elevated lysosomal pH, ATP13A2 might be involved in organellar acidification (Dehay et al., 2012b; Tsunemi and Krainc, 2014). However, whether the lysosome would require additional H^+^ pumps is questionable, as lysosome acidification primarily depends on the activity of V-type ATPases, which are highly efficient and abundant lysosomal H^+^ pumps. V-type ATPases also work together with Cl^−^ channels for generating the steep H^+^ gradient in the lysosome (Marshansky and Futai, 2008).

#### Can the ATP13A2 TM sequence support ion transport?

Establishing conclusively that ATP13A2 is a cation transporter will depend on a biochemical characterization of the purified pumps reconstituted in lipid vesicles that allow for measurements of transport. So far, we can only question whether the ATP13A2 protein supports Zn^2+^, Mn^2+^, Mg^2+^, or H^+^ transport. As explained before, the M4 region in P-type ATPases is critically involved in substrate coordination and specific M4 sequence motifs correlate well with substrate specificity. We first compared the sequence of ATP13A2 with SPCA, an established P-type Mn^2+^ transport ATPase (Figure 4). M4 of ATP13A2 (PPALP) strikingly differs from the SPCA M4 region (PEGLP). The Glu residue that coordinates Mn^2+^ and serves as a gating residue is absent in ATP13A2 and is replaced by a hydrophobic residue that is difficult to reconcile with ion binding. However, ion-coordination in SPCA not only depends on M4, but also involves oxygen atoms of the peptide backbone and polar residues positioned on M5 and M6 (Vangheluwe et al., 2009). In ATP13A2, at least one conserved negatively charged residue is found in M6 and conserved polar residues are found on M4, M5 and M6, which in theory could support ion coordination (Figure 4) (Sorensen et al., 2010). Notably, critically conserved Asp residues in the M6 segment of the P3 plasma membrane H^+^-ATPase (Buch-Pedersen et al., 2009) and the P2 Ca^2+^-ATPases (Toyoshima et al., 2000) play a role in respectively H^+^ coordination and Ca^2+^ coordination.

Coordination of Zn^2+^ typically involves His and/or Cys residues (Simonson and Calimet, 2002). Also in the P-type ATPase heavy metal transporters, conserved Cys residues are found in the M4 region (a CPC motif in the CopA copper transporter) (Gourdon et al., 2011). No obvious conserved His, Cys or even a Met are found in the TM region of ATP13A2, questioning whether coordination of Zn^2+^ may occur. However, a striking similarity is observed between ATP13A2 and the P1B heavy metal P-type ATPases, which both have extra N-terminal helices in common (Sorensen et al., 2010; Gourdon et al., 2011). In the CopA copper transporter, the N-terminal platform recruits metal-chaperones for copper delivery (Gourdon et al., 2011). Whether metal chaperones would bind ATP13A2 remains to be tested. Several putative ATP13A2 interactors were identified with a split-ubiquitin yeast two hybrid screening (Usenovic et al., 2012b) and several genetic interactions for *YPK9* were identified in yeast (Chesi et al., 2012), but no obvious metal chaperones or proteins involved in metal transport were found.

In conclusion, at least some residues in the M-region of ATP13A2 might support ion binding, but the overall composition significantly differs from other P-type Mn^2+^ and Zn^2+^ transporters. So until strong biochemical evidence becomes available, we may need to consider other possibilities.

### Is ATP13A2 a late endosomal/lysosomal flippase?

Among the P-type ATPases the 14 members of the P4 ATPases transport other ligands than inorganic cations. Members of this subfamily flip phospholipids from one membrane leaflet to the other. Because P5 ATPases phylogenetically are more related to P4 ATPases than to any other P-type ATPase subfamily (Figure 1) (Axelsen and Palmgren, 1998), it may be interesting to consider the possibility that P5 ATPases likewise might be involved in lipid or organic ion transport. The structural requirements of P4-type flippases are only just emerging and candidate residues have been identified that are important for lipid transport (Coleman et al., 2012; Baldridge and Graham, 2013; Baldridge et al., 2013). An Ile residue in the conserved P4 sequence motif on M4 (PISL) appears important for the binding and translocation of the phospholipid and shows functional analogy to a conserved Glu in M4 of P2-type ATPases that in these pumps serve as a gating residue (Vestergaard et al., 2014). At this point it remains hard to say whether P5B-type ATPases fulfill the sequence requirements of a typical flippase. Remarkably, a Pro in the M4 motif of the P5 is found at the position of the conserved Glu in P2 and Ile in P4.

Amongst other possible hypotheses, we should consider the possibility that ATP13A2 might be a flippase that transports a lipid or another organic molecule from one membrane leaflet to the other. Such an activity might in turn control vesicular dependent processes that regulate ion homeostasis, exosome formation, mitophagy/autophagy and α-synuclein clearance.

As a putative (lipid) flippase, ATP13A2 might alter membrane curvature, alter lipid dynamics, organize lipid microdomains or expose/remove important signaling molecules at one or the other membrane leaflet (Graham, 2004; Palmgren and Nissen, 2011), which might for instance regulate α-synuclein membrane interactions. Moreover, ATP13A2 is implicated in mitochondrial clearance and exosome formation at the site of the late endosome, MVB and lysosome. A putative (lipid) flippase might here be strategically important as these organelles undergo continuous vesicle forming and vesicular fusion events to deliver, sort or remove cargo. This might require a tight regulation of membrane dynamics. At the end station for autophagsome delivery, ATP13A2 might control the fusion process of the autophagosomes. ATP13A2 might for instance compose a fusion-compatible lipid microdomain or expose important signaling molecules required for fusion. Alternatively, changes in the lysosomal lipid distribution may regulate autophagy pathways, which are known to be sensitive to changes in the lipid environment (Ferguson et al., 2009; Rodriguez-Navarro et al., 2012). ATP13A2 might regulate micro-autophagy, a process depending on membrane invagination to take up cargo for degradation. It might also be involved in the formation of intraluminal vesicles of the MVBs which impacts on α-synuclein removal (Kong et al., 2014).

## Conclusion

Although the cell biological context in which ATP13A2 is involved is gradually emerging, studying the molecular function and substrate specificity of ATP13A2 using biochemical methods and isolated systems will be required to unravel the substrate specificity and transport properties of ATP13A2. Understanding ATP13A2 at the molecular level will reveal its link to KRS, NCL, dystonia, and PD. This might open new therapeutic possibilities to treat this spectrum of disorders.

### Conflict of interest statement

The authors declare that the research was conducted in the absence of any commercial or financial relationships that could be construed as a potential conflict of interest.

## Supplementary material

Supplemental Table 1 **Genetic interactions of *YPK9* in *S. cerevisiae* obtained from the yeast genome database (http://yeastgenome.org)**. The data were were classified using BioGrid (http://thebiogrid.org) according to organellar distribution and function. A summary is provided in Figure 5.
